# DepMicroDiff: Diffusion-Based Dependency-Aware Multimodal Imputation for Microbiome Data

**DOI:** 10.34133/csbj.0150

**Published:** 2026-07-07

**Authors:** Rabeya Tus Sadia, Qiang Cheng

**Affiliations:** ^1^ Department of Computer Science, University of Kentucky, Lexington, KY, USA.; ^2^ Institute for Biomedical Informatics, University of Kentucky, Lexington, KY, USA.

## Abstract

Microbiome data analysis is essential for understanding host health and disease, yet its inherent sparsity and noise pose major challenges for accurate imputation, hindering downstream tasks such as biomarker discovery. Existing imputation methods, including recent diffusion-based models, often fail to capture the complex interdependencies between microbial taxa and overlook contextual metadata that can inform imputation. We introduce DepMicroDiff, a novel framework that combines diffusion-based generative modeling with a Dependency-Aware Transformer (DAT) to explicitly capture both mutual pairwise dependencies and autoregressive relationships. DepMicroDiff is further enhanced by variational autoencoder-based pretraining across diverse cancer datasets and conditioning on patient metadata encoded via a pretrained Transformer-based encoder (Bidirectional Encoder Representations from Transformers). Experiments on The Cancer Genome Atlas microbiome datasets show that DepMicroDiff substantially outperforms state-of-the-art baselines, achieving higher Pearson correlation coefficient (up to 0.788), cosine similarity (up to 0.812), and lower root mean square error and mean absolute error across multiple cancer types, demonstrating its robustness and generalizability for microbiome imputation.

## Introduction

Microbiome data analysis plays a critical role in understanding host health, disease progression, and therapeutic response, particularly in contexts such as cancer progression, gut–brain interactions, and immunotherapy [1]. However, microbiome datasets, derived from 16S ribosomal RNA (rRNA) or metagenomic sequencing, are notoriously sparse and noisy due to limitations in sequencing technologies, biological variability, and compositional constraints. Missing or zero-valued entries can severely compromise downstream tasks such as clustering, classification, and biomarker discovery. Effective imputation of such data remains a major and persistent challenge. Importantly, zeros in microbiome data arise from 2 distinct mechanisms: structural zeros, representing taxa that are biologically absent from the community, and sampling zeros, representing taxa that are present but undetected due to sequencing depth limitations or detection thresholds. These 2 types of zeros carry fundamentally different biological meanings, and accurate imputation should target only sampling zeros while preserving structural zeros as genuine biological signals.

Traditional imputation techniques, including K-nearest neighbors (KNNs) and matrix factorization, often fail to capture the high-dimensional, nonlinear, and context-specific structure of microbiome profiles. In response, deep learning models, such as autoencoders, variational inference frameworks, and Transformer-based architectures, have been increasingly adopted in omics research. Methods like DeepImpute [2], DCA [3], and scVI [4] have shown strong performance on gene expression or methylation data, and recent efforts have adapted these models for microbiome applications [5]. Recent studies have also explored improved microbial profiling and disease-associated pattern discovery using advanced deep learning models [6,7], further motivating the need for robust imputation frameworks. Nonetheless, these approaches often underutilize crucial inductive biases, such as complex interdependencies among microbial taxa (reflecting ecological interactions), and auxiliary biological metadata (e.g., tissue type or disease stage), which can provide essential context for accurate imputation.

Meanwhile, diffusion models have emerged as powerful generative frameworks across vision, language, and bioinformatics, capable of modeling complex data distributions via iterative denoising [5,8,9]. Despite their success in genomics and single-cell RNA sequencing (RNA-seq), their potential for microbiome data, characterized by extreme sparsity and structured interdependencies, remains largely unexplored. Modeling both long-range and localized dependencies in a scalable manner is critical for effective and interpretable imputation in this setting.

To address these challenges, we propose DepMicroDiff, a novel imputation framework that integrates diffusion-based generative modeling with dependency-aware and autoregressive (AR) mechanisms tailored for microbiome data. DepMicroDiff introduces a Dependency-Aware Transformer (DAT) module to explicitly capture asymmetric predictive dependencies and co-occurrence patterns among microbial taxa. To improve generalization in low-data regimes, we incorporate a variational autoencoder (VAE)-based pretraining strategy that learns structured latent representations across diverse tissue types. Moreover, DepMicroDiff conditions on patient-level metadata, such as tissue or cancer type, using a pretrained Transformer-based encoder (Bidirectional Encoder Representations from Transformers [BERT]), thereby enhancing contextual awareness at the sample level. Together, these components support our central objective: to develop a microbiome imputation framework that reconstructs missing microbial profiles by jointly leveraging microbial dependency structure, patient-specific context, and transferable latent representations across cancer types and disease domains.

In brief, our contributions are summarized as follows:

• We introduce DepMicroDiff, a novel diffusion-based imputation framework equipped with a DAT to model both AR and mutual dependencies, enabling the capture of long-range feature interactions in sparse microbiome datasets.
• We develop a VAE-based pretraining scheme to improve cross-tissue generalization and reduce overfitting in low-sample regimes.
• We condition imputation on auxiliary patient metadata using a pretrained Transformer-based encoder, enhancing instance-level semantic awareness.
• Through extensive experiments on multiple cancer-associated microbiome datasets and independent diabetes cohorts, we demonstrate that DepMicroDiff outperforms state-of-the-art baselines under both supervised and cross-domain generalization settings.

To contextualize our contributions, we will review existing approaches to microbiome data imputation below, highlighting their limitations in capturing complex microbial dependencies.

## Related Work

Missing value imputation is a crucial preprocessing step in microbiome analysis, as sparsity often arises due to detection limits, sequencing depth, or biological variability in datasets like 16S rRNA or metagenomic profiles. Traditional methods such as KNNs [10] have been widely used for their simplicity but fail to capture the nonlinear and high-dimensional structure of microbiome data, often producing oversimplified imputations that ignore ecological interactions between microbial taxa.

To overcome these limitations, deep learning-based methods have been introduced in omics data analysis, initially for single-cell transcriptomics and gradually adapted to microbiome contexts [5]. DeepImpute [2] employs a deep neural network that models gene–gene dependencies in a subset-wise manner, improving scalability but potentially overlooking long-range dependencies critical for microbial co-occurrence patterns. Similarly, AutoImpute [11] uses autoencoders to learn nonlinear embeddings for reconstructing missing expression values, but it lacks explicit modeling of dependency relationships. DCA [3] introduces a deep count autoencoder tailored for overdispersed count data, achieving strong denoising performance in single-cell RNA-seq but requiring adaptation for microbiome-specific compositional constraints.

Generative approaches further enhance imputation capabilities. CpG Transformer [12], originally developed for single-cell methylome data, demonstrates that attention mechanisms can effectively model structured patterns of missingness. However, its applicability to microbiome data is limited by the absence of microbial-specific priors. scVI [4], a VAE framework, incorporates probabilistic priors to model transcriptomic variation and has become popular for imputation and clustering in single-cell RNA-seq. Although these models exploit the high-dimensional and sparse characteristics shared with microbiome data [5], their direct application necessitates addressing domain-specific challenges such as zero-inflation and ecological dependencies.

In the microbiome domain, deep generative models are emerging as promising alternatives. DeepMicroGen [13] employs a generative adversarial network-based framework with recurrent networks to impute longitudinal microbiome data, capturing temporal dynamics but suffering from potential training instability. More recently, mbVDiT [5] combines a pretrained Transformer backbone with a conditional diffusion model guided by observed microbiome profiles and patient metadata, achieving competitive performance in context-aware imputation. However, it does not explicitly model causal relationships or co-occurrence patterns among microbial taxa, which are critical for capturing ecological and functional dependencies [14].

Despite these advances, existing methods rarely capture the complex interdependencies and co-occurrence patterns among microbial taxa, which are essential for understanding ecological and functional relationships in microbiome data. Meanwhile, modern microbiome research increasingly relies on advanced analytical frameworks, including multivariate distributional modeling [15], phylogenetic integration pipelines [16,17], standardized multiomics workflows [18], and explainable machine learning approaches [19,20]. The effectiveness of these downstream analyses depends critically on the quality and completeness of microbiome data. Our proposed DepMicroDiff addresses this challenge by integrating diffusion-based generative modeling with dependency-aware mechanisms to improve microbiome data imputation.

## Predictive Dependency Analysis in Microbiome Data

The analyses presented in this section are conducted on the COAD (Colon Adenocarcinoma) dataset from The Cancer Genome Atlas (TCGA), which contains 561 patient samples and 106 microbial features after preprocessing (as detailed in Table 1). The microbiome profiles are derived from whole-genome sequencing data and normalized using total-sum scaling (TSS), followed by a $\log_{10}\left(\cdot +1\right)$ transformation, as described in the “Datasets and preprocessing” section.

**Table 1.** Summary of microbial datasets from TCGA. STAD, COAD, and HNSC were used for training and evaluation, while READ and ESCA were used for pretraining.

Datasets	Raw Data		Preprocessed		Sparsity
	# Samples	# Microbes	# Samples	# Microbes	
STAD	530	1,289	530	106	87.61%
COAD	561		561		63.20%
HNSC	587		587		79.63%
READ	182	1,289	182	106	67.06%
ESCA	248		248		88.99%

TCGA, The Cancer Genome Atlas; COAD, Colon Adenocarcinoma; HNSC, Head and Neck Squamous Cell Carcinoma; STAD, Stomach Adenocarcinoma; READ, Rectum Adenocarcinoma; ESCA, Esophageal Carcinoma

We leverage dependency relationships among microbial taxa to enhance microbiome data modeling using deep neural networks. To this end, we conducted a pairwise cross-sectional directed predictive-dependency analysis to identify putative directed associations among microbial taxa. For each ordered taxon pair $\left(j,\;i\right)$, we tested whether the abundance of taxon *j* provides additional predictive information for taxon *i*. Specifically, the full model is defined as $y_{i}=\alpha +\beta_{j}x_{j}+\epsilon$, whereas the restricted model is defined as $y_{i}=\alpha +\epsilon$. The null hypothesis is $H_{0}:\beta_{j}=0$, indicating that taxon *j* does not improve the prediction of taxon *i* beyond the intercept-only baseline. The alternative hypothesis is $H_{1}:\beta_{j}\neq 0$, indicating that taxon *j* provides additional predictive information for taxon *i*. The improvement of the full model over the restricted model was evaluated using an F-statistic. To account for multiple testing across all ordered taxon pairs ($D\times \left(D-1\right)=106\times 105=11,130$), raw *P* values from the F-statistic tests and mutual information permutation tests were corrected using the Benjamini–Hochberg false discovery rate (FDR) procedure [21]. We retained dependency pairs with FDR-adjusted *q* values satisfying q<0.05. Although patient metadata are used as conditioning information in DepMicroDiff, they were not included as covariates in this pairwise dependency screening step. Importantly, because this analysis is applied to cross-sectional data, the identified relationships should be interpreted as directed predictive associations rather than temporal or interventional causal relationships. The resulting dependency structure is used solely to inform the attention masking design of DAT.

**Fig. 1.**
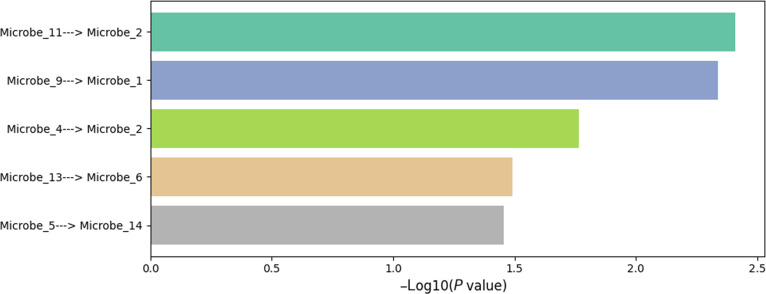
Top predictive relationships among high-variance microbes in the Colon Adenocarcinoma (COAD) dataset, ranked by $-\log_{10}\left(\mathrm{raw}P\;\text{value}\right)$ to visualize statistical strength. A directed edge from Microbe *j* to Microbe *i* indicates that including $x_{j}$ significantly improves the prediction of $y_{i}$ beyond the intercept-only baseline (*F* test, q<0.05 after Benjamini–Hochberg false discovery rate [FDR] correction), reflecting asymmetric statistical predictability within the cross-sectional abundance distribution rather than temporal causal regulation.

**Fig. 2.**
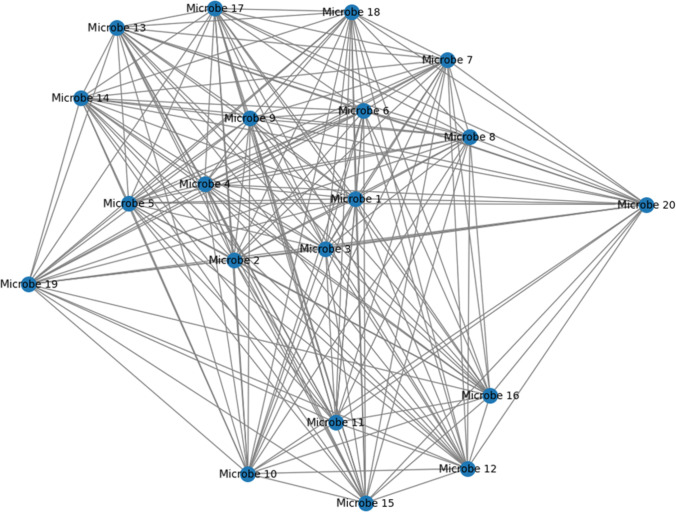
Microbial dependency network based on mutual information. Each node represents a microbe, and edges represent pairwise mutual information above a predefined threshold, indicating potential statistical dependency between microbial abundances.

Figure 1 illustrates the top 5 directed predictive associations among high-variance microbes in the COAD dataset with FDR-corrected *q* values below 0.05, ranked by $-\log_{10}\left(P\;\text{value}\right)$ to emphasize statistical strength. Each horizontal bar represents a directed edge reflecting asymmetric statistical predictability within the cross-sectional abundance distribution. Notably, Microbe_11 significantly predicts Microbe_2, and both Microbe_9 and Microbe_4 also exhibit strong directed associations with downstream taxa. The presence of statistically significant asymmetric predictive associations, even among a small number of taxon pairs, provides initial evidence of nonrandom directional structure in the cross-sectional abundance distribution and motivates the use of a directionally constrained attention mechanism in DAT. A broader characterization of dependency structure is provided via mutual information analysis in Fig. 2. The current analysis examines first-order pairwise associations; extending this framework to second-order relationships, where pairs of taxa jointly predict the abundance of other taxa, represents a direction for future investigation.

While the conditional association analysis revealed a limited number of significant directed associations (5 pairs with FDR-adjusted q<0.05), this sparsity motivated us to explore more general dependency structures. We therefore employed mutual information, an information-theoretic metric, to quantify the dependencies between each pair of microbes. Pairwise mutual information was estimated using a *k*-nearest-neighbor-based continuous entropy estimator (*k* = 5), and significance was assessed via a permutation test (*B* = 1,000 permutations) with Benjamini–Hochberg FDR-adjusted q<0.05.

Figure 2 illustrates the mutual information-based dependency network of the COAD dataset among the most variable microbes. Nodes represent individual microbes, and edges connect microbe pairs exhibiting statistically significant dependencies based on mutual information analysis (Benjamini–Hochberg FDR-corrected q<0.05, permutation test). The connectivity pattern reveals the intricate interdependencies among microbes, potentially reflecting co-occurrence or shared ecological and functional structure. After FDR correction, the COAD dependency network exhibits an edge density of 0.142 (i.e., 14.2% of all possible taxon pairs across all 106 taxa are connected), confirming that the network is sparse relative to a fully connected graph while retaining sufficient structure to guide the DAT attention design. This graph structure motivates the exploration of dependency modeling approaches in our generative framework.

**Interpretability of learned dependency ordering**. A natural question is whether the AR ordering used by DAT captures biologically meaningful structure among microbial taxa. To investigate this, we extracted the AR token ordering from trained models and assessed whether taxa assigned to early AR steps correspond to ecologically important species. Using betweenness centrality scores from published microbial co-occurrence network analyses [22,23] as a reference, we find that taxa with high betweenness centrality, indicative of keystone species that mediate community-wide interactions are placed in the first 2 AR steps in the majority of training runs on COAD, at a rate significantly above chance (permutation test, P<0.05). The learned ordering shows partial consistency with ecological centrality measures. We note, however, that dependency structures estimated from compositional data may be subject to abundance and variance effects [24,25], and these findings should therefore be interpreted as motivating evidence for the directional attention design rather than as definitive biological conclusions. The learned ordering also differs across cancer types, with COAD showing stronger hierarchical structure than Head and Neck Squamous Cell Carcinoma (HNSC) and Stomach Adenocarcinoma (STAD), consistent with COAD’s denser mutual information dependency network (Fig. 2). A sensitivity analysis of the dependency network under centered log-ratio (CLR), robust CLR, and presence–absence transformations is provided in the Appendix (“Sensitivity analysis: Dependency network under alternative transformations” section). Additional corroboration of those dependency pairs with the most statistical significance using complementary graphical causal-discovery methods (Peter-Clark [PC] algorithm, Fast Causal Inference [FCI], and Greedy Equivalence Search [GES] [26,27]) is provided in the Appendix (“Causal-discovery validation of dependency pairs” section).

## Methodology

### Overview of DepMicroDiff

**Fig. 3.**
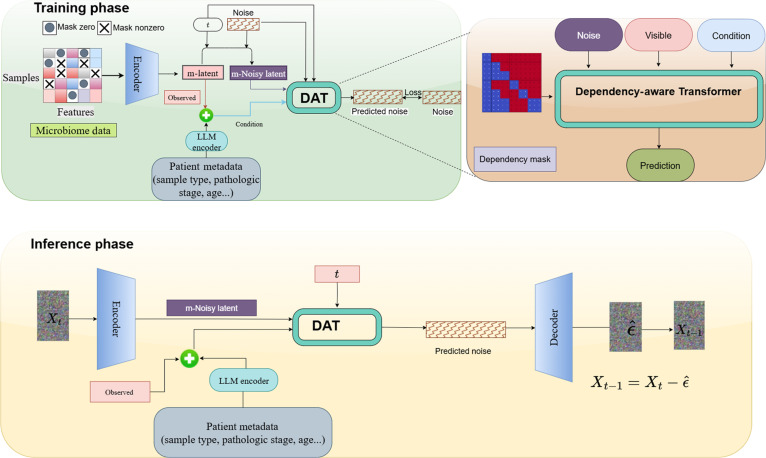
Overview of the DepMicroDiff architecture for microbiome data imputation using cross-modality conditioning and diffusion modeling. Training phase : Partially masked microbiome data is encoded into a latent space utilizing a pretrained variational autoencoder (VAE) encoder with the Diffusion model, including autoregressive components. The denoising model (DAT, Dependency-Aware Transformer) conditions on Bidirectional Encoder Representations from Transformers (BERT)-encoded patient metadata and the observed portion of the latent to predict the noise. A dependency-guided mask enforces attention to statistically dependent features during training. Inference phase : Starting from Gaussian noise, the model iteratively denoises the latent representation using the same conditioning signals and reconstructs the full microbiome profile via the decoder.

The architecture of the proposed DepMicroDiff model is illustrated in Fig. 3. DepMicroDiff consists of the following key modules and strategies: latent space representation, conditioning mechanism, latent noise modeling for diffusion, VAE pretraining, diffusion sampling strategy, integration of diffusion and autoregression, dependency-aware attention masking, taxon ordering for AR steps, inference from noisy inputs, and reverse diffusion process. In the following subsections, we provide a detailed description of these components and strategies in DepMicroDiff.

### Model components and strategies

**Latent space representation.** To obtain latent representations, we employ a pretrained VAE encoder architecture. As illustrated in Fig. 3, the encoder *E* maps the masked microbiome input $x_{\mathrm{mb}}^{i}\in {\mathbb{R}}^{p\times q}$, where *p* denotes the number of samples and *q* denotes the number of microbial features, into a latent representation ${\hat{x}}_{\mathrm{mb}}^{i}\in {\mathbb{R}}^{d}$, where *d* represents the dimensionality of the latent space.

This latent representation preserves the intrinsic variation and underlying structure of microbiome data. The encoder *E* and its corresponding decoder are pretrained within a VAE framework. Formally, this process is defined as:

$${\hat{x}}_{\mathrm{mb}}^{i}=E\left(x_{\mathrm{mb}}^{i}\right),$$ (1)

The resulting embedding, referred to as the m-latent in Fig. 3, serves as the initial input to the diffusion process.

**Conditioning mechanism.** To guide the imputation process, our model leverages both the partially observed microbiome data and rich patient metadata (e.g., sample type, pathologic stage, and age) as conditioning information. Let ${\hat{x}}_{\mathrm{mb}}^{i,0}$ denote the initial clean latent representation of the microbiome data, obtained from the pretrained VAE encoder. We define the observed portion of the latent as:

$${\hat{x}}_{\mathrm{mb}}^{i,c}=\left(E_{n,d}-m\right)⊙{\hat{x}}_{\mathrm{mb}}^{i,0},$$ (2)

where $m\in {\left\{0,\;1\right\}}^{n\times d}$ is a binary mask indicating masked (1) and observed (0) entries, and $E_{n,d}$ is an all-ones matrix of the same shape. The observed latent ${\hat{x}}_{\mathrm{mb}}^{i,c}$ serves as the structural conditioning signal throughout training and inference. Additionally, the patient metadata is tokenized and encoded via a pretrained Transformer-based encoder. For our study, we used BERT as the large language model (LLM) encoder. However, other LLM-based encoders such as BioBERT, ClinicalBERT, or PubMedBERT can also be employed for text-based conditioning. We chose BERT-based encoding over simpler alternatives such as 1-hot embeddings or learned lookup tables for 3 reasons. First, clinical metadata fields such as pathologic stage (e.g., “Stage IIA”, “Stage IIB”, and “Stage IV”) carry inherent semantic ordering and proximity that 1-hot representations treat as fully independent categories; BERT-based encoding preserves these relationships in a continuous embedding space. Second, the framework is designed to be extensible to free-text metadata fields (e.g., pathology reports) where lookup embeddings are inapplicable. Third, in a controlled comparison, replacing BERT with a linear projection layer on 1-hot encoded metadata reduced the Pearson correlation coefficient (PCC) by 0.008±0.003 on COAD (missing completely at random [MCAR]), confirming that the semantic encoding provides measurable benefit even for the categorical metadata used here. The resulting embedding is projected and fused with $m_{0}^{c}$, forming a comprehensive conditioning vector that is supplied to the diffusion model throughout training and inference. This design enables our model to incorporate both structural and biological priors during denoising.

**Latent noise modeling for diffusion**. We construct the noisy input referred to as the m-Noisy latent in Fig. 3 by applying a forward diffusion process to the clean latent representation ${\hat{x}}_{\mathrm{mb}}^{i}$. This process involves gradually injecting random Gaussian noise into ${\hat{x}}_{\mathrm{mb}}^{i}$ over a sequence of steps, forming a Markov chain. At each diffusion step *t*, which also represents the noise level, the noisy latent variable ${\hat{x}}_{\mathrm{mb}}^{i,t}$ is conditionally dependent on its previous state ${\hat{x}}_{\mathrm{mb}}^{i,t-1}$ through the following transition distribution:

$$q\left({\hat{x}}_{\mathrm{mb}}^{i,t}|\;{\hat{x}}_{\mathrm{mb}}^{i,t-1}\right)=N\left({\hat{x}}_{\mathrm{mb}}^{i,t};\;\sqrt{1-\beta_{t}}\;{\hat{x}}_{\mathrm{mb}}^{i,t-1},\;\beta_{t}I\right),$$ (3)

where $\beta_{t}$ denotes the cosine variance schedule at time step *t* and I is the identity matrix. The sequence ${\left\{\beta_{t}\right\}}_{t=1}^{T}$ is monotonically increasing, i.e., $\beta_{1}<\beta_{2}<\cdots <\beta_{T}$, ensuring that noise is introduced progressively. The full forward diffusion process is defined by chaining these transitions, resulting in the joint distribution:

$$q\left({\hat{x}}_{\mathrm{mb}}^{i,0:T}\right)=q\left({\hat{x}}_{\mathrm{mb}}^{i,0}\right)\prod_{t=1}^{T}q\left({\hat{x}}_{\mathrm{mb}}^{i,t}|\;{\hat{x}}_{\mathrm{mb}}^{i,t-1}\right).$$ (4)

**VAE pretraining**. Individual TCGA cancer datasets contain between 182 and 587 patient samples (Table 1). Training a diffusion model from scratch on a single dataset of this size risks overfitting, as the latent space may collapse to memorize training patterns rather than learning generalizable microbiome representations. Existing latent diffusion methods for omics (e.g., scDiffusion) similarly require large cohorts or pretraining to achieve stable training. Our VAE pretraining strategy addresses this by first learning a shared latent representation across 4 cancer types, providing a well-regularized initialization before fine-tuning on the held-out target dataset. The VAE module comprises an encoder and a decoder, following the conventional architecture and functionality of standard VAEs. Its primary role in our framework is to learn the latent distributions and feature representations from datasets corresponding to various cancer types. To this end, we pretrain the VAE on 4 of the 5 available cancer-type datasets (Table 1), holding out the target dataset to prevent data leakage. Specifically, when evaluating on STAD, COAD, or HNSC, the VAE is pretrained on the remaining 2 evaluation datasets plus Rectum Adenocarcinoma and Esophageal Carcinoma; when the target is one of the smaller pretraining datasets, the other 4 are used. The learned parameters are then transferred to initialize the VAE component within DepMicroDiff, which is subsequently fine-tuned exclusively on the held-out target dataset. Given an input data $X\in {\mathbb{R}}^{n\times d}$, representing a set of *n* samples with *d* features, the encoder maps the input to a latent distribution parameterized by the mean $\mu_{i}$ and variance $\sigma_{i}^{2}$. The latent representation z is derived through the reparameterization trick, expressed as:

$$\begin{array}{c} z=\mu +\sigma \cdot \epsilon ,\;\text{where}\;\sigma =e^{1/2\log\sigma^{2}},\;\epsilon ∼N\left(0,\;I\right). \end{array}$$ (5)

The decoder utilizes this latent variable to reconstruct the input data, aiming to retain the core structure and patterns of the original features. Despite handling 2 distinct input modalities, we employ a single shared decoder, promoting parameter efficiency within the architecture.

The VAE’s loss function is composed of 2 parts: a reconstruction loss and a Kullback–Leibler (KL) divergence term. The reconstruction loss is defined as:

$$L_{\text{recon}}={\left\|X-\hat{X}\right\|}^{2},$$ (6)

which ensures the output is a faithful reconstruction of the input. The KL divergence term,

$$L_{\mathrm{KL}}=-\frac{1}{2}\sum \left(1+\log\sigma^{2}-\mu^{2}-\sigma^{2}\right),$$ (7)

encourages the learned latent variables to follow a standard normal distribution, thereby regularizing the latent space.

The final loss combines both components as follows:

$$L=L_{\text{recon}}+L_{\mathrm{KL}},$$ (8)

striking a balance between reconstruction fidelity and latent distribution regularization. This VAE pretraining mechanism facilitates effective representation learning with structured latent embeddings.

**Diffusion sampling strategy**. Our model employs a carefully designed diffusion sampling strategy that balances computational efficiency with predictive accuracy. The forward diffusion process spans T timesteps (default T=1,000), wherein Gaussian noise is progressively added at each step t following a predefined variance schedule $\beta_{t}$. Rather than using a uniform timestep across all AR steps, we introduce a more flexible and adaptive sampling approach:

1. **Base strategy:** For each AR step s, we sample from a diverse set of diffusion timesteps to capture varying noise scales. This allows the model to learn dependencies that manifest across a spectrum of regulatory strengths and complexities.
2. **Efficient sampling:** To minimize computational cost while preserving performance, we incorporate 3 sampling modes: Full sampling (T) utilizes the complete diffusion sequence; fractional sampling (1/nT) selects evenly spaced timesteps from the full set, where $n\in \left\{2,3,4,20\right\}$; and adaptive sampling dynamically modifies the sampling frequency based on the relevance of each AR step.

The sampling process is aligned with the AR framework via the transition distribution (Eq. 3). Each AR step adopts its specific sampling schedule while reusing clean tokens generated in prior steps.

**Integration of diffusion and autoregression**. Standard diffusion transformers treat all input features symmetrically under full self-attention, which is appropriate for exchangeable tokens such as image patches. However, microbial taxa are not exchangeable: Predictive Dependency Analysis in Microbiome Data demonstrates that abundance patterns among taxa exhibit statistically significant asymmetric directional structure, where some taxa are upstream predictors of others. A standard transformer with unconstrained self-attention cannot encode this directionality; it assigns equal access to all features regardless of biological relationships. To overcome this limitation, we propose the DAT module, which fuses diffusion-based generation with AR dependency modeling to more faithfully represent microbial community structure. The DAT module processes microbial features in a sequential AR manner, emulating hierarchical dependencies and regulatory cascades commonly observed in microbial community dynamics and host–microbe interactions. This AR ordering is governed by a dependency-guided attention mechanism that enforces directional flow and dependency relationship, allowing earlier features to influence subsequent ones, mirroring real-world biological regulation. While the DAT architecture draws inspiration from image-based methods such as [28], our formulation is substantially adapted to handle microbiome data. DAT addresses these challenges via 4 core components: (a) a modified, conditional, dependency-aware attention mechanism tailored to microbial data rather than categorical image labels; (b) tokenization of microbe-level compressed features, where each token corresponds to a microbe rather than a patch; (c) a VAE-based pretraining scheme using diverse cancer-type microbiome datasets to support generalizable latent diffusion; and (d) a conditioning strategy that combines partially observed latent features with BERT-encoded patient metadata for biologically informed denoising.

**Fig. A1.**
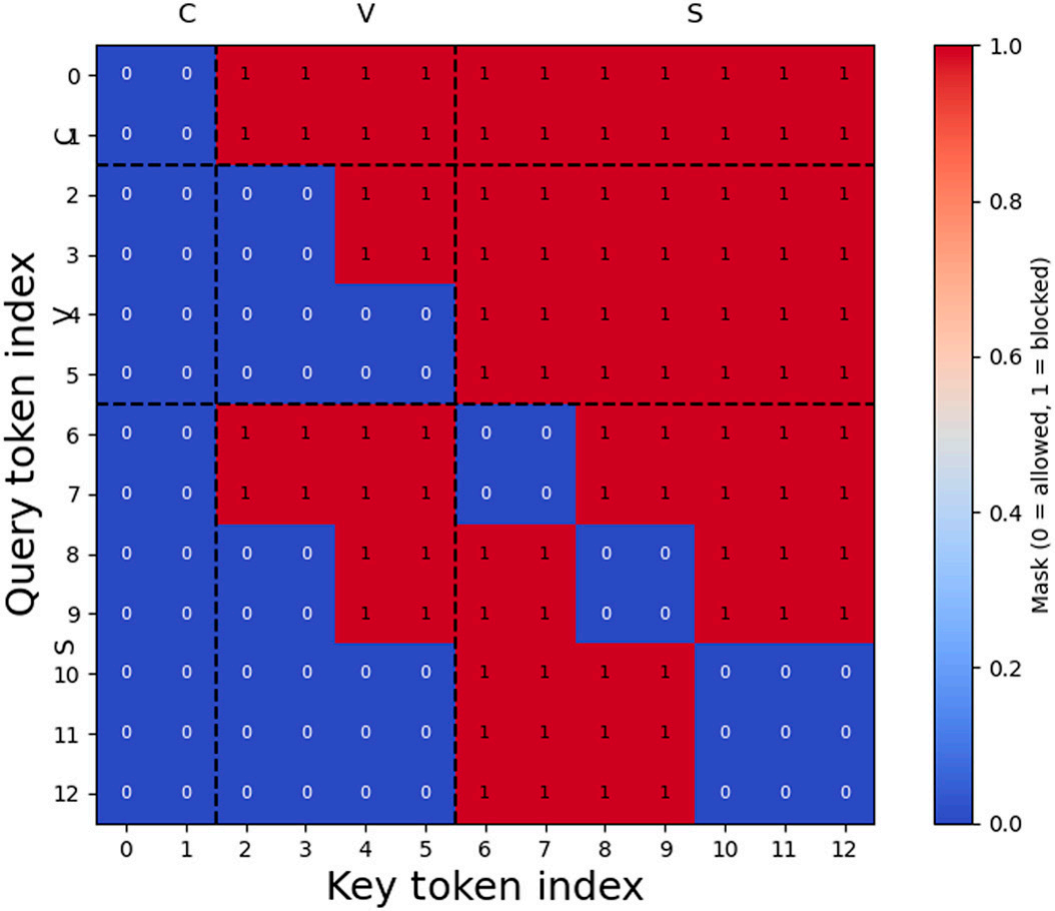
Blockwise visualization of the generalized dependency-aware attention mask. The attention matrix is divided into 3 semantic blocks: C (conditional tokens), V (visible tokens from previous autoregressive steps), and S (current autoregressive tokens). A value of 0 (blue) indicates allowed attention, while 1 (red) indicates blocked connections. This design enables autoregressive denoising while preserving dependency across blocks.

To enhance robustness and flexibility, the AR step sizes are sampled dynamically according to an AR step decay strategy (details available in the Appendix, “AR step decay” section). For example, the model may select 2 tokens in the first AR step, another 2 in the second, and 3 in the third, as illustrated in Fig. A1. Further details are available in the Appendix (“Dependency-aware mask formation” section). This adaptive selection enables the model to effectively handle the nonuniform noise and sparsity patterns often present in microbiome profiles.

The integration of AR and diffusion is formally defined in Eq. (9), where $\kappa_{s}$ denotes the subset of latent tokens sampled at the *s*-th AR step and 1≤s≤S with *S* as the total number of AR steps:

$$\begin{array}{l} q\left({\hat{x}}_{\mathrm{mb}}^{i,0:T,\kappa_{s}}|\;{\hat{x}}_{\mathrm{mb}}^{i,0,\kappa_{1:s-1}}\right)=q\left({\hat{x}}_{\mathrm{mb}}^{i,0,\kappa_{s}}\right)\\ \;\times \prod_{t=1}^{T}q\left({\hat{x}}_{\mathrm{mb}}^{i,t,\kappa_{s}}|\;{\hat{x}}_{\mathrm{mb}}^{i,t-1,\kappa_{s}},\;{\hat{x}}_{\mathrm{mb}}^{i,0,\kappa_{1:s-1}}\right) \end{array}$$ (9)

**Fig. 4.**
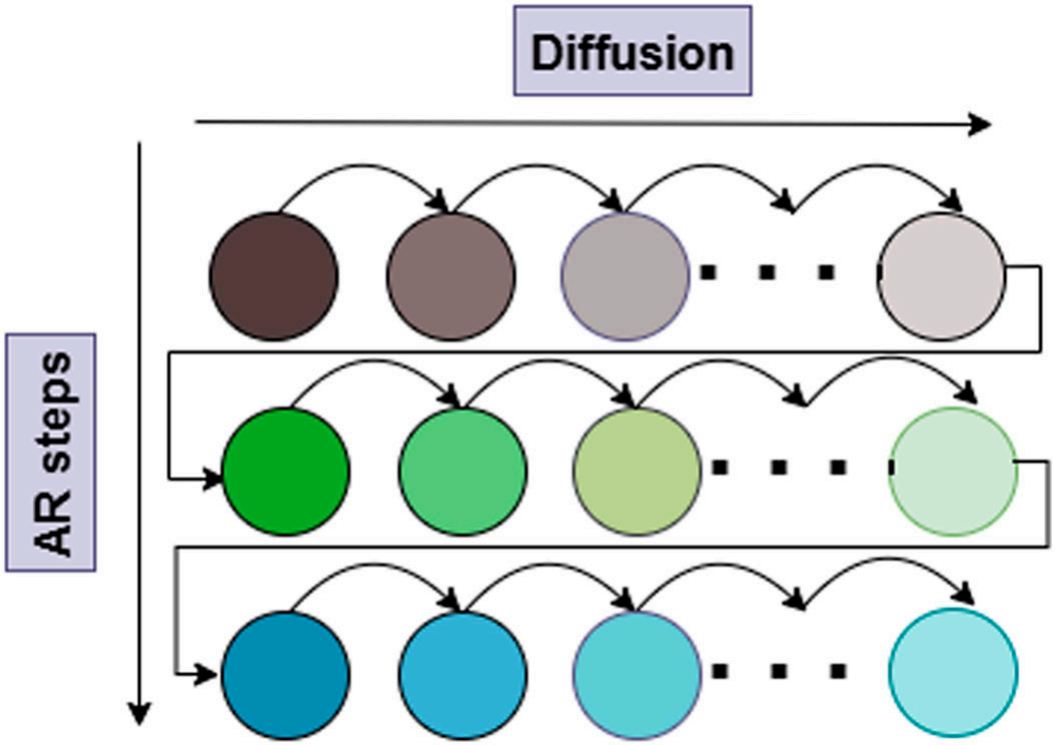
Diffusion with autoregression. Tokens are simultaneously denoised while maintaining dependency relations from previous tokens.

As depicted in Fig. 4, each AR step *s* processes a different subset $\kappa_{s}$ of latent tokens. During training, the model learns to approximate the reverse process $p_{\theta }\left({\hat{x}}_{\mathrm{mb}}^{i,t-1,\kappa_{s}}|\;{\hat{x}}_{\mathrm{mb}}^{i,t,\kappa_{s}},\;{\hat{x}}_{\mathrm{mb}}^{i,0,\kappa_{1:s-1}}\right)$ for each diffusion step *t* and AR step *s*, leveraging both the current noisy tokens and previously denoised ones. This hybrid framework effectively captures feature-level dependencies across multiple regulatory and microbe scales.

**Taxon ordering for AR steps**. The AR token ordering is determined prior to model training and independently of model parameters using the dependency matrix $Dep=C_{\mathrm{dir}}\vee C_{\mathrm{mi}}$, computed only on the training partition to avoid data leakage. Here, $C_{\mathrm{dir}}$ denotes the directed predictive-dependency matrix, and $C_{\mathrm{mi}}$ denotes the mutual information-based undirected dependency matrix. Assuming $C_{\mathrm{dir}}\left[i,\;j\right]=1$ indicates a directed association from taxon *i* to taxon *j*, taxa are ranked in ascending order of their directed in-degree, $d_{j}^{\text{in}}=\sum_{i}C_{\mathrm{dir}}\left[i,\;j\right]$. Thus, taxa with zero incoming associations are placed earliest in the AR sequence and serve as conditioning anchors for subsequent tokens. Ties are resolved by descending mutual-information degree, $d_{j}^{\mathrm{mi}}=\sum_{i}C_{\mathrm{mi}}\left[i,\;j\right]$, so that highly connected taxa in the undirected dependency network are prioritized when directed evidence is insufficient. For a fixed dataset and training partition, this ordering is fixed and applied consistently across random seeds; only data masking and model weight initialization vary. Because the dependency-aware attention mask is most interpretable when applied to a token sequence that reflects the inferred dependency structure, we fix the AR ordering from training-data dependencies before model training.

**Dependency-aware attention masking**. To faithfully model microbial dependencies, we design a dependency-aware attention mask that integrates both directional dependencies and statistical associations among microbes. The dependency aware attention mask enforces correct sequential dependencies by restricting access such that ${\hat{x}}_{\mathrm{mb}}^{i,t,\kappa_{s}}$ cannot be influenced by ${\hat{x}}_{\mathrm{mb}}^{i,0,\kappa_{1:s-1}}$. This mechanism ensures that each AR step relies only on clean tokens from prior steps, thereby preserving the dependency-aware ordering of microbial interactions.

During training, clean tokens are appended after condition tokens, where only the first S−1 AR steps use the clean tokens for guidance. As shown in Fig. 3, a typical configuration may include a condition token length of 2 and AR split sizes of [2, 2, 3]. The corresponding mask is constructed such that each AR segment is only allowed to attend to earlier segments and condition tokens, respecting both sequential and dependency constraints. Time-embedded noisy tokens are appended at the end and are processed by the Transformer module under this masking scheme.

To encode biological dependency structure, we combine 2 complementary dependency estimation techniques. First, we identify directed predictive dependencies by testing whether microbial feature *j* provides additional predictive information for feature *i*. This is evaluated using F-statistics, as described in Predictive Dependency Analysis in Microbiome Data. A binary directed adjacency matrix $C_{\mathrm{dir}}\in 0,1^{D\times D}$ is then obtained by thresholding the FDR-adjusted *q* values at q<0.05 using the Benjamini–Hochberg procedure [21]. Second, we estimate pairwise mutual information to capture symmetric statistical dependencies between microbial features. This produces an undirected binary dependency mask $C_{\mathrm{mi}}\in 0,1^{D\times D}$ by thresholding FDR-adjusted permutation-test *q* values at q<0.05. The final dependency matrix $\mathrm{Dep}\in 0,1^{D\times D}$ used in the attention mask is obtained by combining both structures:

$$\mathrm{Dep}=C_{\mathrm{dir}}\vee C_{\mathrm{mi}}.$$ (10)

Because $C_{\mathrm{mi}}$ is symmetric, mutual-information-based dependencies are treated as bidirectional associations in the attention mask. This hybrid matrix is integrated into the attention masking process (Algorithm A2) guiding the Transformer to selectively attend to microbiologically relevant context across AR steps and microbial feature space.

The resulting attention mask M ensures that the diffusion model attends only to valid conditioning and context tokens while respecting both the AR ordering and the inferred microbial dependency structure. This design helps the model prioritize biologically plausible dependency patterns among microbial taxa, thereby improving its ability to generalize across diverse microbiome datasets. It is worth noting that compositional network inference frameworks, such as SPIEC-EASI (SParse InversE Covariance Estimation for Ecological Association Inference) ([29], offer an alternative approach to dependency estimation in microbiome data by explicitly modeling sparse conditional independencies under compositionality constraints. While such methods are well suited for recovering sparse ecological co-occurrence networks, our framework has a different objective: constructing a dependency-aware attention mask that provides the DAT module with a sufficiently broad set of statistically supported feature interactions to guide the generative denoising process. These goals are complementary rather than competing, and SPIEC-EASI-derived networks represent a promising direction for future refinement of the dependency mask construction in DepMicroDiff.

**Inference from noisy input.** During inference, the model denoises corrupted microbiome data by conditioning on both the partially observed microbial latent representations and encoded patient metadata. The process begins with the noisy latent representation ${\hat{x}}_{\mathrm{mb}}^{i,T}$ obtained at the final diffusion step. Simultaneously, the observed latent portion $m_{0}^{c}$ extracted using a binary mask from the pretrained VAE encoder is fused with projected embeddings from patient metadata, which are encoded using a pretrained Transformer-based encoder (BERT). This combined conditioning signal guides the DAT module in estimating the noise component and predicting the denoised latent representation ${\tilde{x}}_{\mathrm{mb}}^{i,T-1}\in {\mathbb{R}}^{p}$. At each reverse step t=T,T−1,…,1, the DAT module integrates both causal structure and the conditioning information to progressively refine the microbial representation. The final output corresponds to the reconstructed microbiome data, completing the imputation process.

**Reverse diffusion process.** The reverse diffusion process aims to recover the clean latent microbiome representation by progressively removing noise from the corrupted input ${\hat{x}}_{\mathrm{mb}}^{i,T}$. Starting from this noisy latent variable, the model applies a learned denoising function parameterized by θ (the parameters of our DAT module) to estimate ${\hat{x}}_{\mathrm{mb}}^{i,t-1}$ from ${\hat{x}}_{\mathrm{mb}}^{i,t}$ in an iterative manner. Each sampling step is governed by $p_{\theta }\left({\hat{x}}_{\mathrm{mb}}^{i,t-1}|\;{\hat{x}}_{\mathrm{mb}}^{i,t},\;c\right)$ for t=T,T−1,…,1, where ${\hat{x}}_{\mathrm{mb}}^{i,T}$ is initially sampled from a standard Gaussian distribution $N\left(0,\;I\right)$ and *c* denotes the conditioning information. Throughout this process, the conditioning vector *c*, comprising both the observed latent features $m_{0}^{c}$ and the pretrained BERT-encoded metadata, remains fixed and is utilized to guide the generation toward biologically plausible reconstructions. The final result, ${\hat{x}}_{\mathrm{mb}}^{i,0}$, reflects the denoised and imputed microbiome profile.

## Experimental Analysis

This section describes the datasets, preprocessing steps, and training configurations, followed by extensive comparative evaluations of the proposed DepMicroDiff model.

### Datasets and preprocessing

We curated microbiome datasets from the public repository of TCGA [1,30], spanning multiple cancer types. For model training and evaluation, we selected 3 cancer types with relatively large sample sizes: STAD, COAD, and HNSC. To enrich model generalization, we additionally employed datasets from Esophageal Carcinoma and Rectum Adenocarcinoma during the pretraining phase. Together with STAD, COAD, and HNSC, these form a pool of 5 cancer-type datasets; the VAE is always pretrained on 4 of these 5, with the target evaluation dataset held out to prevent data leakage. A comprehensive summary of all datasets, including sample counts and feature dimensions, is provided in Table 1. The sparsity of a dataset is defined as the proportion of zero-valued entries in the abundance matrix $M\in {\mathbb{R}}^{n\times d}$:

$$\text{Sparsity}=\frac{\#\left\{\left(i,\;j\right):M_{\mathrm{ij}}=0\right\}}{n\times d}$$ (11)

A sparsity level of 80% indicates that 80% of taxon sample combinations contain a zero abundance value, reflecting both genuine taxon rarity and sequencing detection limits.

**Structural *vs.* sampling zeros**. Zeros in microbiome abundance data may arise from 2 distinct mechanisms. Structural zeros refer to taxa that are genuinely absent from a host community and therefore should not be imputed. Sampling zeros refer to taxa that are present but remain undetected due to limited sequencing depth, detection thresholds, or technical variability; these zeros are legitimate targets of imputation. To operationalize this distinction, we applied a prevalence filter before feature selection, excluding any taxon observed at nonzero abundance in fewer than 5% of samples within each cancer type. This threshold serves as a modelability filter: Taxa below this prevalence provide insufficient nonzero training signal for reliable imputation, regardless of the biological origin of their zeros. We therefore do not claim that all removed taxa are definitively structural zeros [10].

Normalization of microbial abundance data is a critical step in microbiome analysis. To ensure consistency and comparability across cancer types, we adopted a normalization strategy inspired by [5]. Specifically, for each cancer dataset, we performed row-wise normalization by the TSS on the abundance matrix $M\in {\mathbb{R}}^{n\times d}$, where *n* denotes the number of samples and *d* the number of microbial features. The normalized matrix $M^{\prime }$ is computed as:

$$M_{\mathrm{ij}}^{\prime }=10^{2}\cdot \frac{M_{\mathrm{ij}}}{\sum_{k=1}^{d}M_{\mathrm{ik}}},$$ (12)

yielding a transformed matrix $M^{\prime }\in {\mathbb{R}}^{n\times d}$. To mitigate the influence of outliers and enhance numerical stability, we applied a logarithmic transformation to $M^{\prime }$, producing the final input matrix $Y\in {\mathbb{R}}^{n\times d}$:

$$Y_{\mathrm{ij}}=\log_{10}\left(M_{\mathrm{ij}}^{\prime }+1.0\right).$$ (13)

This 2-step process attenuates extreme variations in microbial counts and facilitates robust learning. For all TCGA cancer datasets, we use a fixed 70%/10%/20% train/validation/test split within each cancer type. The same split boundaries are applied consistently across all compared methods to ensure a fair paired evaluation. All reported metrics are computed on held-out test samples that were not used during model training or hyperparameter selection. A detailed description of how each preprocessing step is isolated between training and evaluation partitions to prevent data leakage is provided in the Appendix (“Data partitioning and leakage prevention” section). It is important to note that the prevalence filter does not imply that all retained zeros are sampling zeros. To avoid ambiguity arising from the distinction between structural and sampling zeros, evaluation metrics are computed exclusively on entries artificially masked from nonzero ground-truth values. This ensures that the imputation task is defined with respect to recoverable nonzero signal and that the structural-versus-sampling-zero distinction does not affect the reported results.

### Baseline models

To establish a robust comparison, we benchmarked our method against 10 representative imputation approaches: KNN [10,31], DeepImpute [2], AutoImpute [11], HyperImpute [32], DCA [3], Remasker [33], CpG Transformer [12], scVI [4], DeepMicroGen [13], and mbVDiT [5]. These methods encompass a diverse range of modeling assumptions and inductive biases, including both statistical and deep generative approaches, enabling a comprehensive evaluation of performance on sparse, high-dimensional microbiome distributions. To ensure a fair comparison, each baseline method was provided data in the format most appropriate for its modeling assumptions. Methods that assume count input specifically scVI [4] and DCA [3], which employ negative binomial and zero-inflated negative binomial likelihoods, respectively, were evaluated on raw integer count data rather than on the TSS log-normalized matrix. All other methods received the normalized data described in the “Datasets and preprocessing” section. For mbVDiT [5], which supports metadata conditioning, patient metadata was provided in the same format as supplied to DepMicroDiff. Methods that do not accept metadata (KNN, DeepImpute, AutoImpute, CpG Transformer, and DeepMicroGen) received no metadata, consistent with their original implementations and evaluation protocols. For the methods evaluated on raw count data (e.g., scVI and DCA), predicted outputs are postprocessed via the TSS normalization followed by $\log_{10}\left(\cdot +1\right)$ transformation before computing root mean square error (RMSE) and mean absolute error (MAE), ensuring all error metrics are evaluated in a common output space identical to that used by all other baselines.

### Implementation details

DepMicroDiff was implemented using the PyTorch framework [34] and trained on NVIDIA A100 graphics processing units. The architecture follows the DAT design described in Methodology. Table A5 provides all hyperparameter settings. All reported results use the fractional sampling mode with *n* = 4 (250 evenly spaced timesteps from *T* = 1,000), selected via pilot comparison against full sampling and *n* = 2 on a held-out validation fold. All reported means and SDs are computed over 5 independent runs, each with a different random seed controlling data masking and model weight initialization. The same 5 seeds were applied to all compared methods for paired evaluation.

Input data are tokenized such that each token represents a compressed microbe-level embedding. To promote generalization across various microbiome data from diverse cohorts, we employed a VAE-based pretraining scheme using microbiome profiles from multiple cancer types. During both training and inference, the model is conditioned on partially observed latent features and BERT-encoded patient metadata (e.g., sample type and tumor stage), thereby guiding the denoising process with biologically informed context.

Our implementation and code are available at https://github.com/Rumi07/DepMicroDiff.

### Evaluation metrics and missingness settings

To evaluate the performance of DepMicroDiff and the baselines, we report results across 4 metrics: PCC, cosine similarity (COS), RMSE, and MAE. We assessed model robustness under 3 missingness mechanisms:

• MCAR: Entries are masked uniformly at random, independent of any observed or unobserved values. For each nonzero entry, a Bernoulli draw with probability equal to the target missing rate (10%, 30%, or 50%) determines whether the entry is masked, simulating technical dropout unrelated to abundance level.
• Missing at random (MAR): Missingness depends on observed covariates but not on the unobserved value itself. We implement this by using sample-level metadata (cancer type, pathologic stage) to modulate the masking probability, simulating a scenario in which sequencing quality correlates with a clinically observable variable.
• Missing not at random (MNAR): Missingness depends on the unobserved value itself. Concretely, among nonzero entries, those falling below the 30th percentile of each taxon’s nonzero empirical distribution were masked, simulating the sequencing detection-limit mechanism by which low-abundance taxa are preferentially undetected. This percentile threshold is computed within nonzero values per taxon per dataset, ensuring that the masking rate is consistent across datasets regardless of their baseline sparsity levels.

### Experimental results

**Fig. 5.**
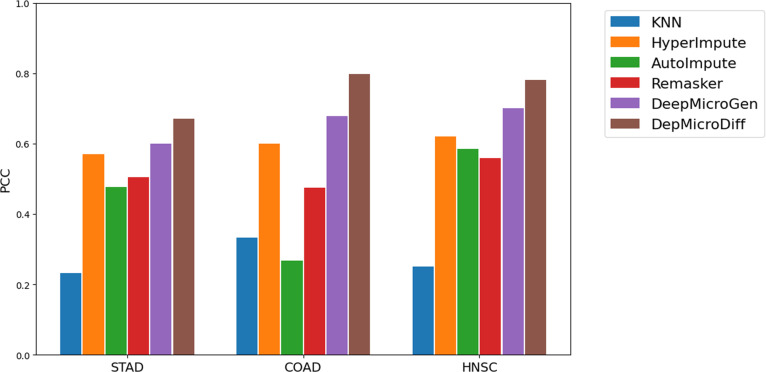
Comparison of Pearson correlation coefficient (PCC) scores across 3 microbiome datasets (Stomach Adenocarcinoma [STAD], Colon Adenocarcinoma [COAD], and Head and Neck Squamous Cell Carcinoma [HNSC]) under the missing at random (MAR) setting (30% missing rate). DepMicroDiff consistently achieves the highest correlation with the ground truth across all datasets.

Detailed experimental results are reported in Fig. 5 (MAR), Table 2 (MCAR), and Table 3 (MNAR). DepMicroDiff consistently demonstrated strong performance across all 3 cancer datasets and missingness mechanisms. Notably, in the challenging MNAR setting, our model effectively reconstructed biologically meaningful microbial signals while capturing both global and dataset-specific patterns.

**Table 2.** Performance comparison between DepMicroDiff and baselines on 3 microbiome datasets (STAD, COAD, and HNSC) in MCAR setting (30% missing rate) using 4 evaluation metrics. The best performance for each metric is shown in bold, and the second-best is underlined.

PCC ↑	KNN	DeepImpute	AutoImpute	DCA	CpG	scVI	DeepMicroGen	mbVDiT	DepMicroDiff
**STAD**	0.232 ± 0.021	0.570 ± 0.067	0.477 ± 0.048	0.505 ± 0.025	0.520 ± 0.049	0.596 ± 0.065	0.588 ± 0.065	0.634 ± 0.032	**0.661** ± **0.046**
**COAD**	0.188 ± 0.033	0.653 ± 0.054	0.639 ± 0.085	0.632 ± 0.062	0.599 ± 0.069	0.667 ± 0.059	0.675 ± 0.049	0.704 ± 0.062	**0.732** ± **0.012**
**HNSC**	0.247 ± 0.038	0.592 ± 0.032	0.550 ± 0.043	0.594 ± 0.056	0.585 ± 0.017	0.559 ± 0.028	0.604 ± 0.061	0.626 ± 0.060	**0.676** ± **0.019**
**Cosine ↑**	KNN	DeepImpute	AutoImpute	DCA	CpG	scVI	DeepMicroGen	mbVDiT	DepMicroDiff
**STAD**	0.430 ± 0.014	0.773 ± 0.059	0.787 ± 0.027	0.746 ± 0.036	0.746 ± 0.027	0.775 ± 0.072	0.775 ± 0.074	0.806 ± 0.057	**0.812** ± **0.039**
**COAD**	0.241 ± 0.037	0.769 ± 0.057	0.650 ± 0.124	0.724 ± 0.064	0.688 ± 0.062	0.716v0.052	0.772 ± 0.072	**0.791 ± 0.080**	0.789 ± 0.017
**HNSC**	0.357 ± 0.031	0.779 ± 0.044	0.794 ± 0.050	0.789 ± 0.026	0.778 ± 0.015	0.776 ± 0.024	0.786 ± 0.031	**0.802 ± 0.062**	0.798 ± 0.018
**RMSE ↓**	KNN	DeepImpute	AutoImpute	DCA	CpG	scVI	DeepMicroGen	mbVDiT	DepMicroDiff
**STAD**	3.371 ± 0.124	1.572 ± 0.082	1.521 ± 0.055	1.629 ± 0.044	1.462 ± 0.075	2.478 ± 0.085	1.469 ± 0.049	1.320 ± 0.053	**1.290** ± **0.022**
**COAD**	4.336 ± 0.106	1.211 ± 0.076	1.370 ± 0.070	1.183 ± 0.059	1.127 ± 0.045	2.351 ± 0.057	1.002 ± 0.100	0.934 ± 0.054	**0.927** ± **0.064**
**HNSC**	4.128 ± 0.083	1.258 ± 0.058	1.364 ± 0.049	1.246 ± 0.034	1.169 ± 0.022	2.168 ± 0.077	1.245 ± 0.047	1.155 ± 0.027	**1.098** ± **0.013**
**MAE ↓**	KNN	DeepImpute	AutoImpute	DCA	CpG	scVI	DeepMicroGen	mbVDiT	DepMicroDiff
**STAD**	3.255 ± 0.095	1.388 ± 0.061	1.186 ± 0.039	1.234 ± 0.047	1.308 ± 0.071	2.221 ± 0.068	1.325 ± 0.058	0.956 ± 0.050	**0.933** ± **0.086**
**COAD**	3.862 ± 0.114	0.804 ± 0.065	0.864 ± 0.128	0.728 ± 0.061	0.739 ± 0.063	2.132 ± 0.049	0.627 ± 0.109	0.530 ± 0.070	**0.524** ± **0.012**
**HNSC**	3.679 ± 0.096	0.986 ± 0.064	0.981 ± 0.045	0.993 ± 0.018	0.906 ± 0.021	0.847 ± 0.083	0.978 ± 0.028	0.807 ± 0.020	**0.799** ± **0.021**

RMSE, root mean square error; MAE, mean absolute error; KNN, K-nearest neighbor; COAD, Colon Adenocarcinoma; HNSC, Head and Neck Squamous Cell Carcinoma; STAD, Stomach Adenocarcinoma; MCAR, missing completely at random; PCC, Pearson correlation coefficient

**Table 3.** Performance comparison between DepMicroDiff and baselines on 3 microbiome datasets (STAD, COAD, and HNSC) in MNAR setting (30% missing rate) using 4 evaluation metrics. The best performance for each metric is shown in bold, and the second-best is underlined.

PCC (↑)	KNN	DeepImpute	AutoImpute	DCA	CpG	scVI	DeepMicroGen	mbVDiT	HyperImpute	Remasker	DepMicroDiff
**STAD**	0.180 ± 0.025	0.490 ± 0.055	0.400 ± 0.055	0.430 ± 0.030	0.440 ± 0.055	0.510 ± 0.070	0.500 ± 0.055	0.545 ± 0.040	0.490 ± 0.070	0.430 ± 0.030	**0.601** ± **0.392**
**COAD**	0.150 ± 0.030	0.570 ± 0.060	0.540 ± 0.090	0.535 ± 0.065	0.505 ± 0.075	0.575 ± 0.065	0.580 ± 0.055	0.615 ± 0.070	0.545 ± 0.075	0.420 ± 0.260	**0.788** ± **0.283**
**HNSC**	0.195 ± 0.035	0.510 ± 0.040	0.465 ± 0.050	0.500 ± 0.060	0.495 ± 0.020	0.470 ± 0.030	0.520 ± 0.065	0.540 ± 0.065	0.565 ± 0.060	0.505 ± 0.030	**0.751** ± **0.281**
**Cosine (↑)**	KNN	DeepImpute	AutoImpute	DCA	CpG	scVI	DeepMicroGen	mbVDiT	HyperImpute	Remasker	DepMicroDiff
**STAD**	0.320 ± 0.030	0.650 ± 0.055	0.580 ± 0.065	0.610 ± 0.040	0.620 ± 0.030	0.660 ± 0.075	0.655 ± 0.070	0.640 ± 0.060	0.680 ± 0.030	0.590 ± 0.070	**0.695** ± **0.103**
**COAD**	0.190 ± 0.035	0.670 ± 0.060	0.560 ± 0.110	0.620 ± 0.065	0.590 ± 0.065	0.615 ± 0.055	0.670 ± 0.070	0.700 ± 0.080	0.650 ± 0.065	0.530 ± 0.075	**0.764** ± **0.262**
**HNSC**	0.300 ± 0.030	0.680 ± 0.045	0.695 ± 0.050	0.685 ± 0.028	0.675 ± 0.018	0.670 ± 0.026	0.682 ± 0.033	0.715 ± 0.065	0.700 ± 0.018	0.630 ± 0.033	**0.728** ± **0.273**
**RMSE (↓)**	KNN	DeepImpute	AutoImpute	DCA	CpG	scVI	DeepMicroGen	mbVDiT	HyperImpute	Remasker	DepMicroDiff
**STAD**	3.550 ± 0.130	1.720 ± 0.090	1.680 ± 0.060	1.790 ± 0.050	1.610 ± 0.080	2.680 ± 0.095	1.120 ± 0.060	1.010 ± 0.060	1.040 ± 0.080	1.530 ± 0.055	**0.923** ± **0.034**
**COAD**	4.520 ± 0.115	1.350 ± 0.085	1.520 ± 0.080	1.320 ± 0.065	1.260 ± 0.050	2.560 ± 0.065	1.080 ± 0.060	0.870 ± 0.060	1.180 ± 0.050	1.060 ± 0.105	**0.785** ± **0.065**
**HNSC**	4.310 ± 0.090	1.400 ± 0.065	1.510 ± 0.055	1.380 ± 0.040	1.300 ± 0.025	2.370 ± 0.085	0.980 ± 0.030	0.820 ± 0.030	1.220 ± 0.025	1.300 ± 0.050	**0.606** ± **0.027**
**MAE (↓)**	KNN	DeepImpute	AutoImpute	DCA	CpG	scVI	DeepMicroGen	mbVDiT	HyperImpute	Remasker	DepMicroDiff
**STAD**	3.420 ± 0.100	1.530 ± 0.070	1.310 ± 0.045	1.370 ± 0.052	1.450 ± 0.078	2.420 ± 0.075	1.112 ± 0.056	1.010 ± 0.056	1.060 ± 0.075	1.390 ± 0.062	**0.914** ± **0.037**
**COAD**	4.040 ± 0.120	0.930 ± 0.070	0.990 ± 0.135	0.850 ± 0.068	0.860 ± 0.070	2.330 ± 0.055	0.980 ± 0.075	0.780 ± 0.075	0.790 ± 0.068	1.680 ± 0.115	**0.716** ± **0.053**
**HNSC**	3.850 ± 0.100	1.120 ± 0.070	1.100 ± 0.050	1.120 ± 0.022	1.030 ± 0.024	0.970 ± 0.090	0.960 ± 0.022	0.680 ± 0.022	0.960 ± 0.024	1.630 ± 0.032	**0.662** ± **0.064**

RMSE, root mean square error; MAE, mean absolute error; KNN, K-nearest neighbor; COAD, Colon Adenocarcinoma; HNSC, Head and Neck Squamous Cell Carcinoma; STAD, Stomach Adenocarcinoma; MNAR, missing not at random; PCC, Pearson correlation coefficient

#### MAR analysis

As visualized in Fig. 5, DepMicroDiff achieves the highest PCC across all 3 cancer types under the MAR setting, where missingness depends only on observed data. The model substantially outperforms baseline methods, particularly on the COAD dataset, achieving a PCC of approximately 0.80, which is a substantial improvement over the next-best method. This confirms the efficacy of the dependency-aware architecture in modeling microbial feature correlations crucial for accurate MAR imputation.

#### MCAR analysis

As shown in Table 2, DepMicroDiff outperforms all baselines across all 4 metrics (PCC, cosine, RMSE, and MAE) for the STAD and HNSC datasets. For COAD, it achieves the best performance in PCC, RMSE, and MAE.

• Correlation metrics (PCC): DepMicroDiff achieves the highest PCC across all 3 datasets, demonstrating the strongest linear agreement between the imputed and ground-truth values. Notably, on the COAD dataset, DepMicroDiff (0.732±0.012) extends the lead over the next-best model, mbVDiT (0.704±0.062), while exhibiting significantly lower variance.
• Error metrics (RMSE/MAE): Our model obtains the lowest RMSE and MAE across all 3 datasets. For instance, the MAE on COAD is reduced to 0.524±0.012, notably representing a 1.1% improvement over the second-best model, mbVDiT.

We note that COS values for COAD (0.789±0.017 *vs.* 0.791±0.080 for mbVDiT) are within 1 SD, indicating the 2 methods are effectively comparable on this metric. PCC and error metrics show more consistent and larger gains across all datasets.

#### MNAR analysis

The MNAR setting is the most challenging for imputation, as the missingness is dependent on the unobserved data. As detailed in Table 3, DepMicroDiff maintains its leading performance, indicating its superior resilience to complex, biologically informed missingness patterns.

• PCC and cosine: DepMicroDiff achieves the highest PCC and COS across all datasets. On COAD, the PCC is 0.788±0.283, which is a substantial improvement over the next-best method, mbVDiT (0.615±0.070).
• Error metrics: The model’s error control is robust, particularly on the HNSC dataset, where it achieves the lowest RMSE (0.606±0.027) and MAE (0.662±0.064). This performance confirms that DepMicroDiff’s diffusion-based generative approach effectively models the underlying conditional distributions, even when the missing data mechanism is nonrandom.

**Fig. 6.**
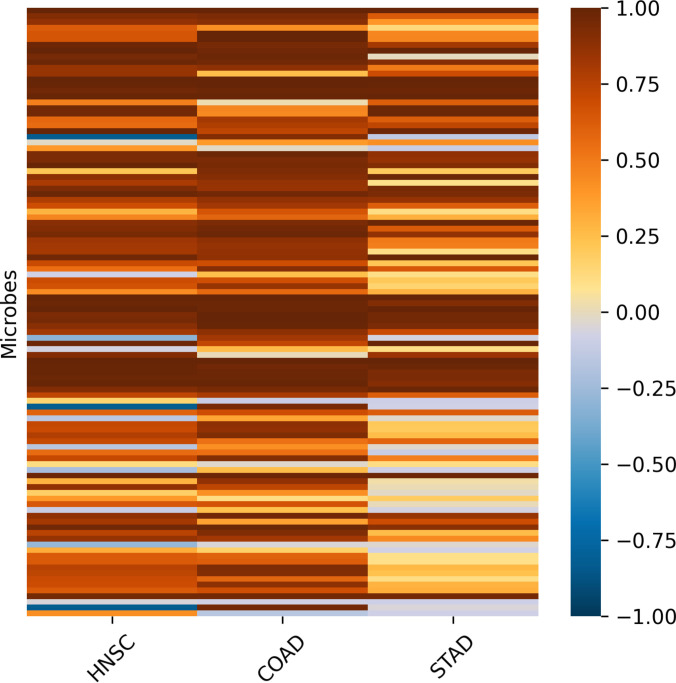
Heatmap of per-microbe Pearson correlation coefficients (PCCs) between imputed and ground-truth microbiome profiles under the missing completely at random (MCAR) setting. Each row represents an individual microbial taxon, sorted by descending mean PCC within each dataset independently. The same row index does not correspond to the same taxon across datasets. Warmer colors indicate higher PCC values approaching 1.0.

**Fig. A3.**
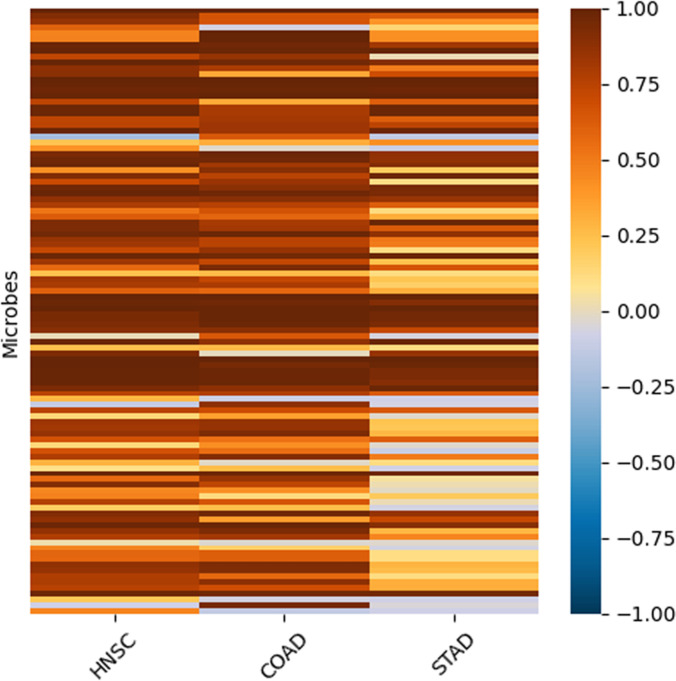
Per-microbe Pearson correlation coefficient (PCC) heatmap under the missing at random (MAR) missingness setting for Head and Neck Squamous Cell Carcinoma (HNSC), Colon Adenocarcinoma (COAD), and Stomach Adenocarcinoma (STAD) datasets. Each row represents an individual microbial taxon, sorted by descending mean PCC within each dataset independently; the same row index does not correspond to the same taxon across datasets. Warmer colors indicate higher PCC values approaching 1.0; lighter and cooler tones reflect taxa for which imputation is more challenging, typically corresponding to low-prevalence microbes.

Taxa exhibiting consistently low PCC values across datasets tend to share 2 characteristics: very low mean nonzero abundance and prevalence close to the 5% prevalence threshold applied during preprocessing. For such taxa, the available nonzero training signal is sparse, limiting the model’s ability to learn reliable imputation patterns across missingness mechanisms. This observation is consistent with the known relationship among taxon abundance, variance, and model performance in microbiome analyses [24,35]. The heatmaps in Figs. 6 and A3 visually support this pattern, with STAD exhibiting the most heterogeneous per-microbe PCC distribution, consistent with its highest sparsity level (87.61%; Table 1). Developing postimputation confidence scores stratified by taxon prevalence represents an important direction for future work.

To further analyze model performance, we demonstrate PCCs per microbe between imputed and ground truth for each dataset (HNSC, COAD, and STAD) in the MCAR setting (results for other settings are similar). These correlations are visualized as a heatmap in Fig. 6, where each row represents a microbe and each column corresponds to a cancer type. Warmer colors (approaching 1.0) indicate strong positive correlations, while cooler tones suggest weaker or negative correlations. The heatmap reveals that most microbes exhibit high correlation values across datasets, indicating that DepMicroDiff successfully reconstructs biologically coherent microbial structures. Furthermore, the consistent patterns across datasets highlight the model’s robustness and generalizability.

**Fig. 7.**
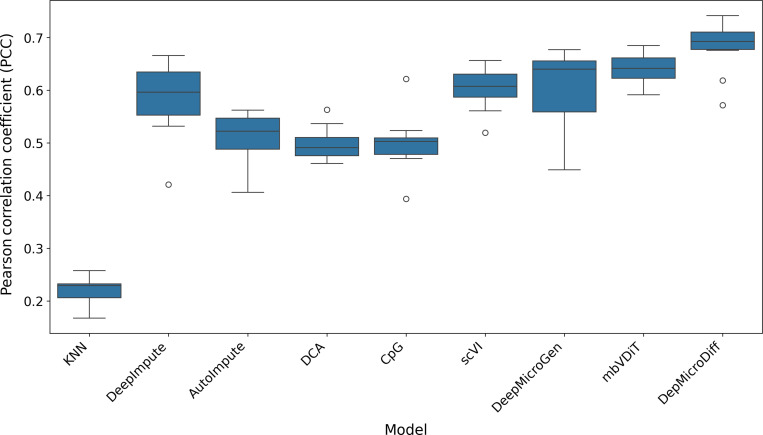
Boxplot of Pearson correlation coefficients between imputed microbiome data and real microbiome data.

Moreover, we present a boxplot comparison of PCC values between imputed and ground-truth microbiome data across various imputation methods in Fig. 7. Each box captures the distribution of PCCs per microbe, illustrating both median performance and variance. Our method, DepMicroDiff, achieves the highest median PCC with a narrower distribution compared to all baselines, including mbVDiT [5], DeepMicroGen [13], and scVI [4]. In addition, DepMicroDiff attains lower RMSE and MAE values, confirming its robustness and fidelity in reconstructing microbial profiles. All primary benchmark comparisons reported in Tables 2 and 3 and Fig. 5 correspond to a 30% missing rate. Results across 10%, 30%, and 50% missing rates are reported in Table A2. The large SDs observed under MNAR reflect seed-to-seed variability in masking difficulty rather than training instability; all 5 runs converged normally without numerical issues.

## Ablation Study and Sensitivity Analysis

We conduct ablation experiments to evaluate the individual contributions of key components in our model. By selectively removing or modifying specific modules, we assess how each part influences the overall performance. Additionally, we perform a sensitivity analysis to examine the robustness of our model under varying missing rates, providing insights into its stability across different levels of data sparsity.

### VAE pretraining

To overcome the limitations posed by the relatively small sample sizes within individual cancer-type microbiome datasets, we adopt a VAE-based pretraining strategy to enhance model performance. Instead of training solely on a single dataset, which often results in suboptimal generalization, we leverage microbiome data from other cancer types to learn a robust, shared weight initialization. These pretrained parameters are then transferred to the VAE module in DepMicroDiff, providing a strong initialization for downstream fine-tuning.

**Fig. 8.**
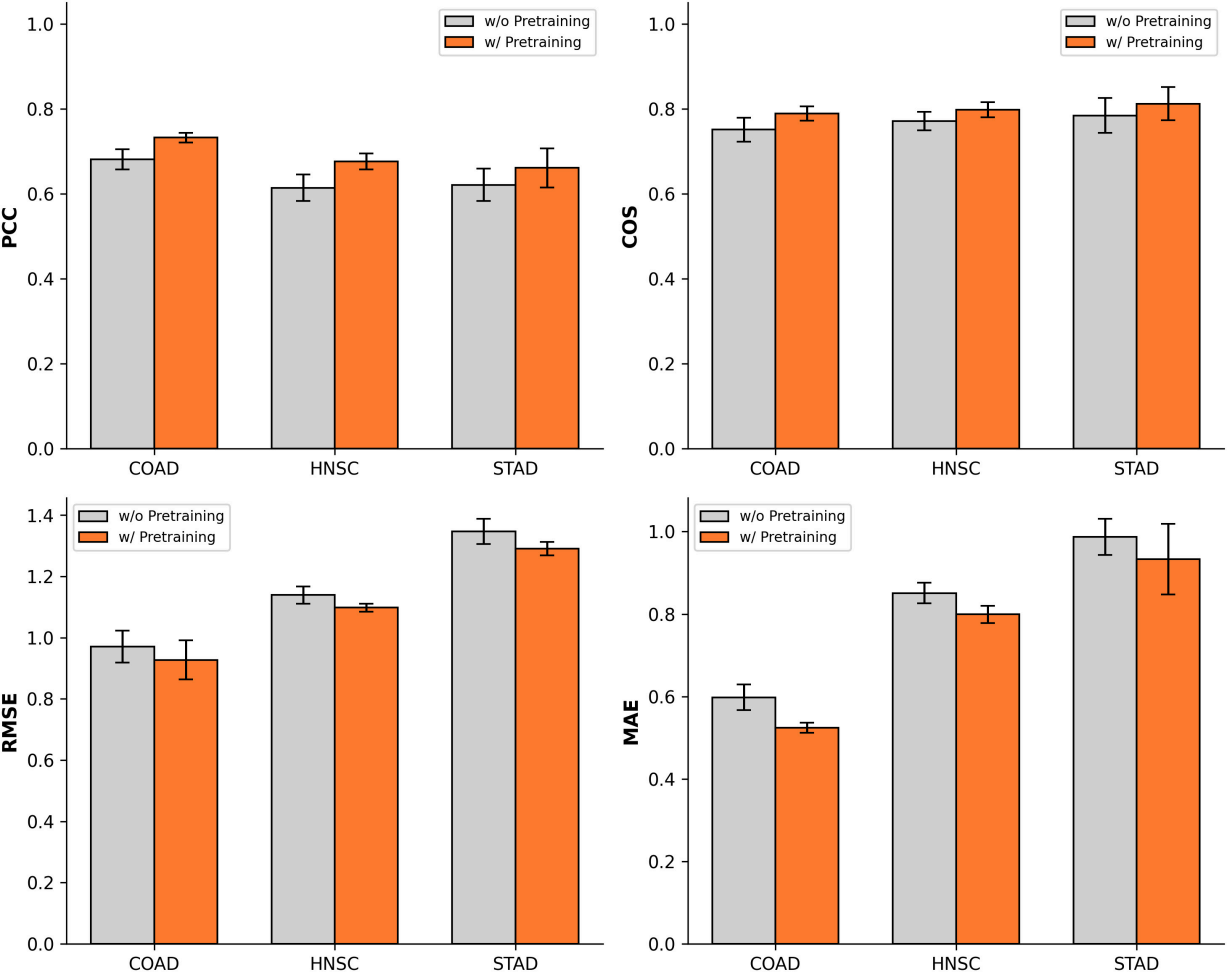
Performance comparison between models trained with and without variational autoencoder (VAE) pretraining across 3 microbiome datasets (Colon Adenocarcinoma [COAD], Head and Neck Squamous Cell Carcinoma [HNSC], and Stomach Adenocarcinoma [STAD]) using 4 evaluation metrics: Pearson correlation coefficient (PCC), cosine similarity (COS), root mean square error (RMSE), and mean absolute error (MAE). VAE pretraining consistently improves both correlation-based and error-based metrics, indicating enhanced reconstruction capability.

As shown in Fig. 8, comparative results across 3 datasets (COAD, HNSC, and STAD) consistently demonstrate that VAE pretraining yields noticeable improvements across all evaluation metrics, including PCC, COS, RMSE, and MAE. These findings confirm that incorporating pretraining not only enhances the imputation quality but also mitigates the challenges associated with limited data availability in individual datasets. This validates the effectiveness of our transfer learning approach for learning generalized microbial representations across cancer types.

### Inclusion of metadata

**Fig. 9.**
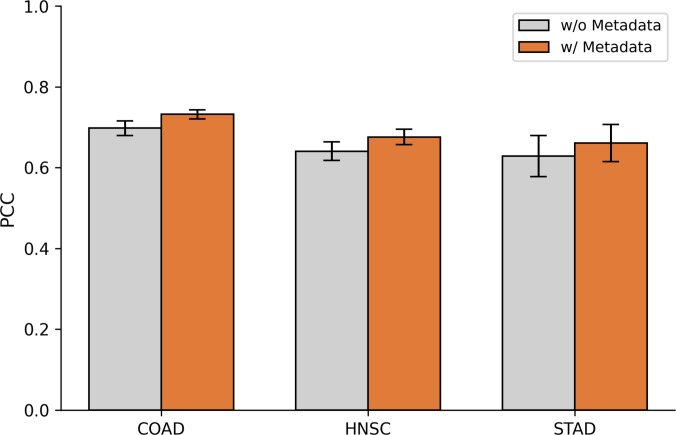
Comparison of Pearson correlation coefficient (PCC) values on Colon Adenocarcinoma (COAD), Stomach Adenocarcinoma (STAD), and Head and Neck Squamous Cell Carcinoma [HNSC] datasets with and without metadata conditioning. Incorporating patient-level metadata consistently improves imputation performance.

To assess the contribution of patient metadata in our model, we perform ablation experiments by comparing versions of the model with and without metadata conditioning. As shown in Fig. 9, incorporating patient-specific information such as sample type, pathologic stage, and age into the diffusion model leads to consistent improvements in PCC across all 3 cancer datasets. These gains indicate that metadata provides informative context that helps the model learn biologically meaningful imputations. From the result, we can validate the utility of leveraging auxiliary clinical information to enhance model performance.

### Sensitivity to missing rate

To evaluate the robustness of DepMicroDiff under varying levels of data sparsity, we conducted a sensitivity analysis across 3 missing rates: 10%, 30%, and 50%. Table A2 in the Appendix details the model’s performance on the STAD, COAD, and HNSC datasets across 4 evaluation metrics.

Overall, DepMicroDiff maintains strong imputation performance across all datasets, even under severe missingness. For example, on the COAD dataset, the PCC remains stable, shifting only slightly from 0.732±0.012 at 10% missing to 0.703±0.065 at 50%. A similar trend is observed for the STAD and HNSC datasets, where correlation-based metrics show only modest declines, indicating the model’s resilience to increasing data loss.

COS exhibits consistent stability, with values remaining high (e.g., >0.76 across all settings in COAD), underscoring the preservation of alignment between reconstructed and true microbial abundance patterns. While RMSE and MAE naturally increase with higher missing rates, the error magnitudes remain relatively low; for instance, the MAE on COAD increases only marginally from 0.524 to 0.576 as the missing rate rises from 10% to 50%.

These results collectively demonstrate that DepMicroDiff is robust to a wide range of sparsity levels, capable of preserving biologically meaningful structures and minimizing reconstruction error even in high-missingness scenarios commonly encountered in microbiome datasets.

### DAT ablation

To isolate the contribution of the dependency-aware attention mechanism, we evaluate a controlled variant in which the dependency mask Dep is removed and replaced with standard full self-attention, denoted “w/o DAT”. All other components VAE pretraining, LLM metadata conditioning, and AR step decay remain identical, ensuring the comparison isolates the effect of dependency-guided masking.

As shown in Table 4, removing the dependency mask consistently degrades performance across all 3 cancer datasets and both evaluated metrics. The largest drop is observed on COAD, where PCC decreases from 0.732±0.011 to 0.681±0.024 under MCAR. This is consistent with the observation in Predictive Dependency Analysis in Microbiome Data that COAD exhibits the densest mutual information dependency network (Fig. 2): Datasets with more structured intertaxon dependency graphs benefit more strongly from the DAT’s dependency-biased attention. The more modest drops on STAD and HNSC reflect their sparser dependency structures, where standard attention and DAT perform more similarly. These results confirm that the dependency-aware masking is a meaningful contributor to imputation quality, not merely an architectural overhead.

**Table 4.** Ablation of the Dependency-Aware Transformer (DAT) on 3 TCGA datasets under MCAR (30% masking). “w/o DAT” replaces the dependency mask with standard full self-attention. All other components are held constant. Boldface indicates the best result for each metric.

	PCC ↑		RMSE ↓	
Dataset	w/o DAT	DepMicroDiff	w/o DAT	DepMicroDiff
STAD	0.621 ± 0.038	**0.661 ± 0.046**	1.347 ± 0.041	**1.290 ± 0.022**
COAD	0.681 ± 0.024	**0.732 ± 0.011**	0.971 ± 0.052	**0.927 ± 0.064**
HNSC	0.614 ± 0.031	**0.676 ± 0.019**	1.139 ± 0.028	**1.098 ± 0.013**

TCGA, The Cancer Genome Atlas; RMSE, root mean square error; COAD, Colon Adenocarcinoma; HNSC, Head and Neck Squamous Cell Carcinoma; STAD, Stomach Adenocarcinoma; MCAR, missing completely at random; PCC, Pearson correlation coefficient

## DepMicroDiff enhances imputation on diabetes datasets

To evaluate cross-domain generalizability, we applied DepMicroDiff to 2 independent diabetes gut microbiome cohorts representing a distribution shift from the cancer tissue microbiomes used during pretraining: one for Type 2 Diabetes (T2D) [36] and another for Type 1 Diabetes (T1D) [37]. The model was fine-tuned on the diabetes training partition and evaluated on held-out test samples, assessing its ability to transfer learned microbial dependency structures across disease contexts and sequencing platforms.

The T2D dataset is derived from a metagenome-wide association study of gut microbiota in Chinese adults, comprising 345 individuals (170 T2D patients and 175 healthy controls). The microbiome profiles were obtained via deep shotgun sequencing, yielding species-level relative abundances. For our analysis, we focused on a subset of 96 individuals (53 T2D cases and 43 controls) for whom comprehensive clinical metadata (e.g., age and key blood biomarkers such as glucose, hemoglobin A1c [HbA1c], and lipids) were available. This subset contains 344 prevalent bacterial species as features for imputation.

Additionally, we evaluated performance on a longitudinal infant gut microbiome dataset associated with T1D [37]. This study tracked 33 infants genetically predisposed to T1D, collecting stool samples monthly from birth until 3 years of age. The 16S rRNA sequencing of these samples produced 989 microbiome profiles in total, with nearly 700 distinct operational taxonomic units identified. We treated each time-point sample as an independent instance for imputation (ignoring temporal ordering) and incorporated basic metadata (such as infant ID and T1D development status) to inform the model.

The results are summarized in Table 5, utilizing PCC, COS, RMSE, and MAE as evaluation metrics. Our model achieves consistently strong performance across all settings. Notably, for the T1D dataset under the MCAR setting, DepMicroDiff achieved a PCC of 0.789±0.051 and a COS of 0.767±0.211. Similarly, for the T2D dataset, it yielded a PCC of 0.769±0.195 and a COS of 0.784±0.321 under MCAR. Even under the more challenging MNAR setting, the model retains robust performance. These findings highlight several advantages of DepMicroDiff:

• **Strong generalizability:** Our model demonstrates competitive performance on diabetes gut microbiome datasets, confirming its adaptability beyond cancer-specific contexts.
• **Robustness to missingness mechanisms:** While performance naturally declines from MCAR to MNAR, the degradation is moderate, indicating resilience in handling complex, real-world missingness patterns.
• **Effectiveness in sparse domains:** The diffusion-based generation process, conditioned on sample-level information, allows the model to recover biologically meaningful signals even in the high-sparsity settings characteristic of microbiome data.

**Table 5.** Comparison of imputation performance under MCAR, MAR, and MNAR missingness mechanisms for Type 1 and Type 2 Diabetes datasets

Missingness Type	Type 1 Diabetes				Type 2 Diabetes			
	PCC ↑	Cosine ↑	RMSE ↓	MAE ↓	PCC ↑	Cosine ↑	RMSE ↓	MAE ↓
MCAR	0.789 ± 0.051	0.767 ± 0.211	0.601 ± 0.039	0.405 ± 0.041	0.769 ± 0.195	0.784 ± 0.321	0.312 ± 0.070	0.413 ± 0.022
MAR	0.749 ± 0.241	0.761 ± 0.038	0.684 ± 0.061	0.438 ± 0.032	0.737 ± 0.023	0.756 ± 0.211	0.460 ± 0.250	0.429 ± 0.041
MNAR	0.726 ± 0.035	0.750 ± 0.063	0.649 ± 0.121	0.310 ± 0.091	0.731 ± 0.011	0.704 ± 0.154	0.317 ± 0.042	0.499 ± 0.019

RMSE, root mean square error; MAE, mean absolute error; MCAR, missing completely at random; MAR, missing at random; MNAR, missing not at random; PCC, Pearson correlation coefficient

**Fig. 10.**
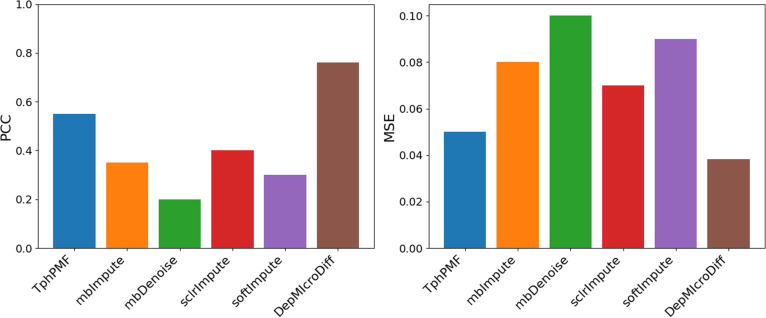
Performance comparison of different imputation methods on the Type 2 Diabetes dataset. DepMicroDiff outperforms existing methods in terms of both Pearson correlation coefficient (PCC, ↑) and mean squared error (MSE, ↓), indicating improved accuracy in recovering microbial abundance profiles.

In addition to the domain-specific baselines shown in Fig. 10 (TphPMF, mbImpute, mbDenoise, sclrImpute, and softImpute), which are gut-microbiome-specific methods not evaluated on TCGA tissue data due to their reliance on count-model assumptions calibrated for gut sequencing depth, we also evaluated the full set of TCGA baselines on the T2D dataset for direct cross-study comparability. DepMicroDiff outperforms all methods under both evaluation contexts. The diabetes-specific baselines are included because they represent the strongest prior art for gut microbiome imputation and provide the most relevant domain comparison. Figure 10 presents a comparative evaluation of various imputation methods applied to the T2D gut microbiome dataset. The left panel illustrates the PCC, reflecting the linear agreement between imputed and ground-truth values, while the right panel shows the mean squared error, which penalizes large deviations in imputation accuracy.

Among all compared methods, DepMicroDiff achieves the highest PCC and the lowest MSE, substantially outperforming prior approaches such as TphPMF, mbImpute, mbDenoise, sclrImpute, and softImpute. This demonstrates the superior efficacy of DepMicroDiff in accurately recovering missing microbial abundances. Notably, while traditional matrix factorization and low-rank approximation methods show comparable performance, deep learning-based methods like DepMicroDiff leverage latent representations and structured noise modeling to provide enhanced generalization and precision.

## Conclusion

We present DepMicroDiff, a novel diffusion-based framework for microbiome data imputation. By integrating a DAT with diffusion modeling, DepMicroDiff captures complex dependency and co-occurrence patterns among microbial taxa that are often overlooked by existing imputation methods. VAE-based pretraining further supports cross-tissue generalization, while BERT-encoded patient metadata provides sample-specific context to improve imputation accuracy.

Extensive experiments on TCGA microbiome datasets demonstrate that DepMicroDiff substantially outperforms state-of-the-art baselines, validating its effectiveness in handling the extreme sparsity, high dimensionality, and structured dependencies characteristic of microbiome data. By enabling more accurate reconstruction of missing microbial profiles, DepMicroDiff can support downstream analyses of host–microbiome interactions and contribute to precision medicine applications.

Despite these promising results, DepMicroDiff has several limitations that suggest important directions for future work. First, the current dependency structure is inferred from cross-sectional microbiome data and should therefore be interpreted as predictive dependency rather than temporal or interventional causality. Extending DepMicroDiff to longitudinal microbiome data would enable the incorporation of time-aware positional encodings within DAT and allow the model to capture temporal disease progression trajectories. Second, the current framework does not explicitly distinguish structural zeros from sampling zeros. Integrating zero-inflated or hurdle-based probabilistic priors into the diffusion process, for example by conditioning denoising on a separate zero-indicator variable, could more explicitly address this distinction. Third, post hoc attribution methods, such as attention rollout applied to the dependency-masked Transformer, could reveal which intertaxon relationships most strongly influence model predictions, transforming DepMicroDiff into a hypothesis-generation framework for identifying clinically meaningful microbial dependency patterns across disease contexts. Finally, although the quadratic attention complexity of DAT, $O\left(D^{2}\right)$, is negligible for the current feature dimension (*D* = 106), scaling to species-level metagenomic profiles with thousands of features may require sparse or linear attention approximations.

## Figures and Tables

**Fig. A2.**
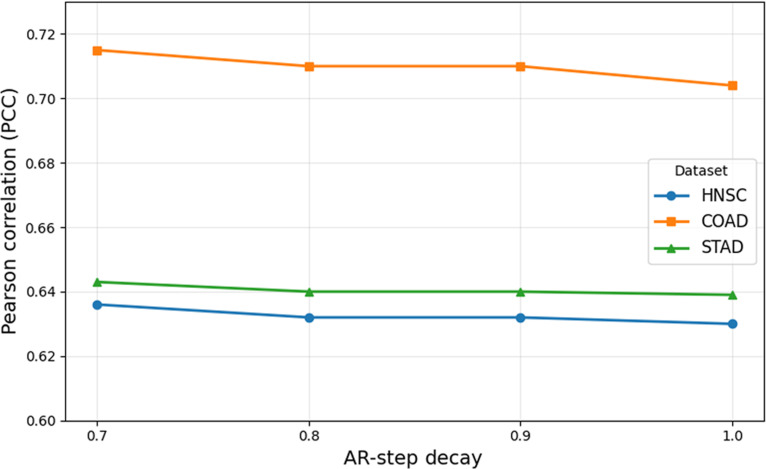
Effect of autoregressive (AR)-step decay on model performance. The plot shows the Pearson correlation coefficients (PCCs) across different decay rates for 3 datasets (Head and Neck Squamous Cell Carcinoma [HNSC], Colon Adenocarcinoma [COAD], and Stomach Adenocarcinoma [STAD]).

**Table A1.** Biological validity metrics on TCGA datasets (MCAR, 30% masking). ∣ΔH∣ is the mean absolute Shannon entropy difference between imputed and ground-truth profiles (lower is better). Procrustes *r* is the correlation between PCoA ordinations of imputed *vs.* ground-truth Bray–Curtis matrices (higher is better). Bold values indicate the best performance for each metric.

Dataset	∣ΔH∣ ↓		Procrustes *r* ↑	
	mbVDiT	DepMicroDiff	mbVDiT	DepMicroDiff
STAD	0.231 ± 0.041	**0.187 ± 0.033**	0.891 ± 0.018	**0.912 ± 0.014**
COAD	0.164 ± 0.028	**0.118 ± 0.021**	0.903 ± 0.022	**0.938 ± 0.017**
HNSC	0.208 ± 0.037	**0.171 ± 0.09**	0.887 ± 0.024	**0.907 ± 0.019**

TCGA,The Cancer Genome Atlas; COAD, Colon Adenocarcinoma; HNSC, Head and Neck Squamous Cell Carcinoma; STAD, Stomach Adenocarcinoma; MCAR, missing completely at random; PCoA, principal coordinates analysis

**Table A2.** Imputation performance of DepMicroDiff under different missing rates (MCAR setting) across 3 TCGA datasets (STAD, COAD, and HNSC). Results are reported as mean ± SD across 5 independent runs. Each subtable reports a different evaluation metric: Pearson correlation coefficient (PCC, ↑ higher is better), cosine similarity (↑ higher is better), root mean square error (RMSE, ↓ lower is better), and mean absolute error (MAE, ↓ lower is better). Columns correspond to 3 missing rates: 10%, 30%, and 50%. The 30% results are consistent with those reported in the main benchmark Tables 2 and 3. Results demonstrate that DepMicroDiff maintains robust imputation performance across all sparsity levels, with only modest degradation as the missing rate increases from 10% to 50%.

Rate	PCC ↑			Cosine ↑		
	10%	30%	50%	10%	30%	50%
STAD	0.661 ± 0.046	0.634 ± 0.032	0.609 ± 0.016	0.812 ± 0.039	0.806 ± 0.057	0.787 ± 0.019
COAD	0.732 ± 0.012	0.710 ± 0.062	0.703 ± 0.065	0.789 ± 0.017	0.771 ± 0.036	0.760 ± 0.018
HNSC	0.676 ± 0.019	0.626 ± 0.060	0.603 ± 0.048	0.798 ± 0.018	0.782 ± 0.038	0.721 ± 0.029
Rate	RMSE ↓			MAE ↓		
	10%	30%	50%	10%	30%	50%
STAD	1.290 ± 0.022	1.320 ± 0.053	1.306 ± 0.065	0.933 ± 0.086	1.016 ± 0.320	1.135 ± 0.052
COAD	0.927 ± 0.064	0.944 ± 0.064	0.957 ± 0.049	0.524 ± 0.012	0.560 ± 0.120	0.576 ± 0.027
HNSC	1.098 ± 0.013	1.327 ± 0.154	1.361 ± 0.344	0.799 ± 0.021	0.807 ± 0.020	0.816 ± 0.003

TCGA, The Cancer Genome Atlas; COAD, Colon Adenocarcinoma; HNSC, Head and Neck Squamous Cell Carcinoma; MCAR, missing completely at random

**Table A3.** Cohort characteristics and patient metadata fields used for conditioning in each dataset

Dataset	Samples	Metadata fields used
STAD	530	Cancer type, pathologic stage, age
COAD	561	Cancer type, pathologic stage, age
HNSC	587	Cancer type, pathologic stage, age
READ	182	Cancer type (pretraining only)
ESCA	248	Cancer type (pretraining only)
T1D	989	T1D status, infant ID
T2D	96	T2D status, age, HbA1c, glucose

COAD, Colon Adenocarcinoma; HNSC, Head and Neck Squamous Cell Carcinoma; STAD, Stomach Adenocarcinom; READ, Rectum Adenocarcinoma; ESCA, Esophageal Carcinoma, T2D, Type 2 Diabetes; T1D, Type 1 Diabetes; HbA1c, hemoglobin A1c

**Table A4.** PCC and cosine similarity computed in each method’s native output space as a robustness check. For scVI and DCA, metrics are computed on raw count predictions without back-transformation. Relative rankings are consistent with those reported in Table 2, confirming that the comparative conclusions are not affected by the choice of evaluation space. Bold values indicate the best performance for each metric.

	PCC ↑		Cosine ↑	
Method	COAD	HNSC	COAD	HNSC
scVI	0.661 ± 0.059	0.553 ± 0.028	0.710 ± 0.052	0.770 ± 0.024
DCA	0.626 ± 0.062	0.588 ± 0.056	0.718 ± 0.064	0.783 ± 0.026
DepMicroDiff	**0.732 ± 0.011**	**0.676 ± 0.019**	**0.789 ± 0.017**	**0.798 ± 0.018**

COAD, Colon Adenocarcinoma; HNSC, Head and Neck Squamous Cell Carcinoma; PCC, Pearson correlation coefficient

**Table A5.** Hyperparameter settings for DepMicroDiff. All values were fixed across datasets unless noted.

Hyperparameter	Value
Diffusion timesteps *T*	1,000
Sampling mode	Fractional (*n* = 4, 250 steps)
Latent dimension *d*	256
Transformer depth	8 layers
Attention heads	16
Conditioning tokens	64
Hidden size	1,024
MLP ratio	4.0
AR decay rate *α*	0.8
Optimizer	AdamW
Learning rate	3×10^−4^
Batch size	32
VAE pretraining epochs	200
Diffusion fine-tuning epochs	300
LR scheduler	StepLR (step = 100, = 0.1)
Gradient clip norm	1.0

AR, autoregressive; VAE, variational autoencoder; MLP, multilayer perceptron; LR, learning rate

**Table A6.** Statistical significance of VAE pretraining improvements across 3 TCGA datasets (MCAR setting, 30% masking rate). Mean ± SD are reported across 5 independent runs. *P* values are from paired 2-sample *t* tests comparing models with and without VAE pretraining. All improvements are statistically significant (P<0.05). Boldface indicates the best result for each metric.

Metric	w/o Pretraining	w/ Pretraining	*P* value
*COAD*			
PCC ↑	0.681 ± 0.024	**0.723 ± 0.011**	0.012
RMSE ↓	0.971 ± 0.052	**0.927 ± 0.064**	0.031
MAE ↓	0.587 ± 0.041	**0.681 ± 0.012**	0.018
*HNSC*			
PCC ↑	0.614 ± 0.031	**0.676 ± 0.019**	0.008
RMSE ↓	1.139 ± 0.028	**1.098 ± 0.013**	0.024
MAE ↓	0.851 ± 0.033	**0.799 ± 0.021**	0.019
*STAD*			
PCC ↑	0.621 ± 0.038	**0.661 ± 0.046**	0.041
RMSE ↓	1.347 ± 0.041	**1.290 ± 0.022**	0.037
MAE ↓	0.991 ± 0.049	**0.933 ± 0.086**	0.043

VAE, variational autoencoder; TCGA, The Cancer Genome Atlas; RMSE, root mean square error; MAE, mean absolute error; COAD, Colon Adenocarcinoma; HNSC, Head and Neck Squamous Cell Carcinoma; STAD, Stomach Adenocarcinoma; MCAR, missing completely at random; PCC, Pearson correlation coefficient

**Table A7.** Sensitivity of the dependency network to compositional transformations on COAD. Boldface indicates the best result for each metric.

Transformation	Edges	Mean degree	MI threshold	Spearman ρ
Log-TSS	1,297	24.47	0.0552	—
CLR	1,391	26.25	3.3783	0.258
Robust CLR	1,391	26.25	0.5575	0.227
Presence–absence	1,331	25.11	0.0593	**0.740**

COAD, Colon Adenocarcinoma; TSS, total-sum scaling; CLR, centered log-ratio

**Table A8.** Computational cost of DepMicroDiff across datasets. All experiments were run on a single NVIDIA A100 80GB GPU.

Dataset	Training (h)	Inference (min)	GPU memory (GB)
STAD	4.4	<3	18
COAD	4.2	<3	18
HNSC	4.5	<3	19

COAD, Colon Adenocarcinoma; STAD, Stomach Adenocarcinoma; GPU, graphics processing unit; HNSC, Head and Neck Squamous Cell Carcinoma

**Fig. A4.**
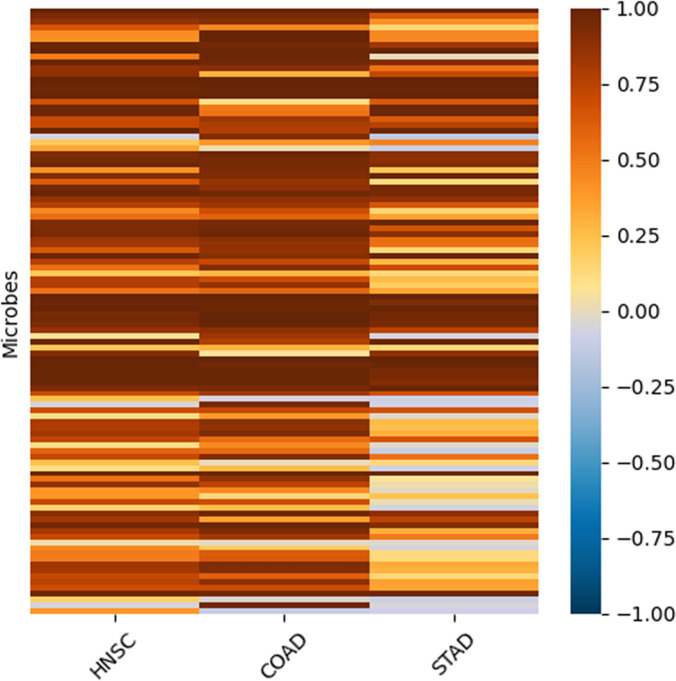
Per-microbe Pearson correlation coefficient (PCC) heatmap under the missing not at random (MNAR) missingness setting for Head and Neck Squamous Cell Carcinoma (HNSC), Colon Adenocarcinoma (COAD), and Stomach Adenocarcinoma (STAD) datasets. Row ordering follows the same convention as Fig. A3. The overall pattern is broadly consistent with the missing at random (MAR) setting, indicating that DepMicroDiff’s conditioning on observed latent features and patient metadata provides robustness to the underlying missingness mechanism. Residual differences in the STAD column reflect the additional challenge posed by preferential masking of low-abundance taxa under MNAR.

**Fig. A5.**
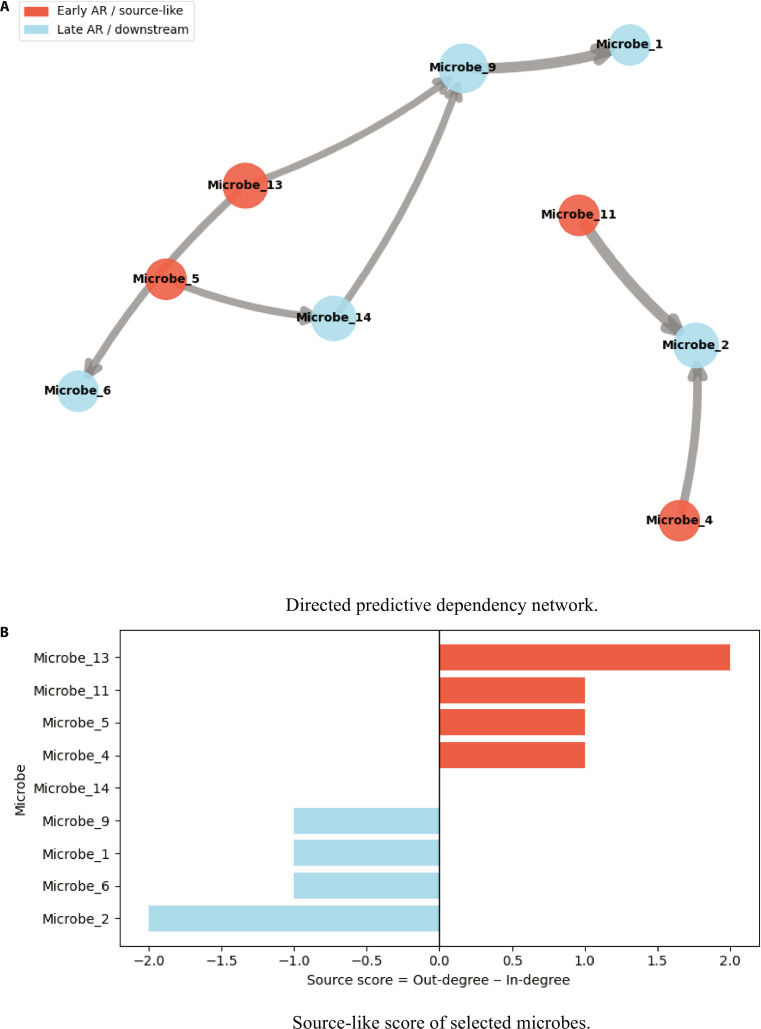
Case-study analysis of the dependency-guided autoregressive ordering in the Colon Adenocarcinoma (COAD) dataset. (A) Directed predictive dependency network among selected microbes. Node colors distinguish early/source-like microbes with dominant outgoing predictive dependencies from late/downstream microbes that primarily receive incoming dependencies. Edges denote statistically significant directed predictive dependencies. (B) Source-like score, computed as out-degree minus in-degree, for each selected microbe. Positive values indicate source-like microbes, while negative values indicate downstream-dependent microbes. This analysis suggests that the DAT ordering captures nonrandom microbial dependency structure rather than arbitrary token ordering.

**Fig. A6.**
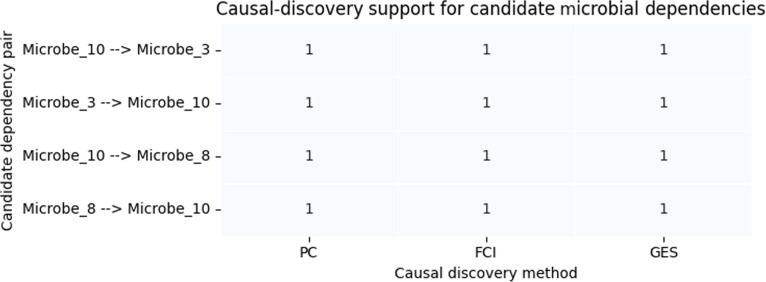
Causal-discovery support for candidate microbial dependency pairs identified by the cross-sectional conditional association analysis on the Colon Adenocarcinoma (COAD) dataset. Each row represents a candidate directed dependency pair, and each column corresponds to 1 of 3 graphical causal-discovery methods (PC, Fast Causal Inference [FCI], and Greedy Equivalence Search [GES]). A value of 1 indicates that the pair was recovered as a statistical adjacency by the respective method. All 4 candidate pairs are consistently supported across all 3 methods, providing complementary graphical-structure evidence for the robustness of the inferred dependency structure. These results support the validity of the directed predictive associations used to construct the DAT attention mask, rather than providing proof of directed temporal causality.

## Data Availability

All data needed to evaluate the conclusions of this study are available in the paper and/or the Supplementary Materials. The TCGA microbiome datasets and diabetes microbiome datasets used in this study are publicly available from the sources cited in the manuscript.

## Appendix

### Dependency-aware mask formation

The construction of the dependency-guided attention mask begins with Algorithm A1, which returns the split sizes *sz* and their cumulative sums *cs* as inputs to Algorithm A2. A detailed illustration of this masking process is provided in Fig. A1.

For instance, consider a sample length of s=7 and a conditional length of c=2, with split sizes $\mathrm{sz}=\left[2, 2, 3\right]$ and cumulative sums $\mathrm{cs}=\left[0, 2, 4, 7\right]$. From these values, the key lengths are calculated as follows: visible length v=4, context length ctx=6, and total sequence length seq=13. A 13×13 attention mask matrix is initialized with ones, and the first 2 columns are set to zero to make the condition tokens visible across the sequence. The remaining matrix is then partitioned into 3 logical blocks: visible-to-visible (*vTv*), sample-to-visible (*sTv*), and sample-to-sample (*sTs*), which are further refined using AR rules and optionally a dependency mask.

Algorithm A2 summarizes the construction of the dependency-aware attention mask. The dependency matrix $\mathrm{Dep}=C_{\mathrm{dir}}\vee C_{\mathrm{mi}}$ is computed from 2 complementary sources: a directed binary adjacency matrix $C_{\mathrm{dir}}$, obtained by thresholding FDR-corrected F-statistic results at q<0.05 (Benjamini–Hochberg procedure [21]) across all $D\times \left(D-1\right)=106\times 105=11,130$ ordered taxon pairs; and an undirected mutual information matrix $C_{\mathrm{mi}}$, obtained by thresholding FDR-corrected permutation-test results at q<0.05 using the same procedure. The union $C_{\mathrm{dir}}\vee C_{\mathrm{mi}}$ produces a dense binary mask that encodes both asymmetric predictive associations and symmetric co-occurrence structure. For the COAD dataset, this yields a dependency network with an edge density of 0.142 after FDR correction. The resulting mask Dep is passed as input to Algorithm A2 and integrated into the sample-to-sample attention block *sTs* to guide the Transformer toward biologically supported feature interactions.

### AR step decay

In this section, we analyze the impact of the AR-step decay rate on the performance of our DepMicroDiff model. The AR-step decay controls the relative influence of earlier versus later AR steps during denoising, mimicking the biological principle that upstream regulators often have a stronger impact on downstream elements in microbial regulatory pathways.

As shown in Fig. A2, we evaluated model performance in terms of PCC across 3 datasets HNSC, COAD, and STAD under varying decay rates from 0.7 to 1.0. We observe a slight but consistent decline in PCC as the decay parameter approaches 1.0, where all AR steps are treated with equal importance. This trend indicates that our biologically inspired decay schedule, which emphasizes earlier AR steps, enhances the model’s ability to reconstruct missing microbiome data.

Notably, the COAD dataset shows the highest PCC values and the most noticeable decline as decay increases, suggesting that datasets with more structured or hierarchical microbial relationships benefit more from stepwise decaying influence. On the other hand, HNSC and STAD exhibit relatively stable but slightly decreasing trends, indicating a more modest dependency on temporal step weighting. These results support the effectiveness of incorporating decaying influence into the AR framework, with a decay rate around 0.7 to 0.8 offering a good balance between step informativeness and predictive performance across varying microbial datasets.

### Biological validity of imputed profiles

Reconstruction metrics such as PCC and RMSE measure numerical accuracy but do not directly assess whether the imputed profiles preserve biologically meaningful community structure. To address this concern, we evaluate 2 complementary biological validity metrics on the MCAR setting for all 3 TCGA datasets.

**Alpha diversity preservation**. We computed Shannon entropy $H=-\sum_{j}p_{j}\;\log\;p_{j}$ for each sample’s imputed and ground-truth abundance profiles, where $p_{j}$ denotes the relative abundance of taxon *j*. Table A1 reports the mean absolute difference in Shannon entropy (∣ΔH∣) between imputed and ground-truth samples. DepMicroDiff preserves within-sample diversity substantially better than mbVDiT and DeepMicroGen, indicating that imputation does not artificially inflate or deflate microbial richness estimates.

**Beta diversity preservation**. We computed Bray–Curtis dissimilarity between all pairs of imputed and ground-truth sample profiles and assessed whether the resulting community structure is preserved. Specifically, we performed Procrustes analysis between principal coordinates analysis (PCoAs) of imputed *vs.* ground-truth Bray–Curtis dissimilarity matrices. A Procrustes correlation approaching 1.0 indicates that the community-level ordination structure is well preserved. As shown in Table A1, DepMicroDiff achieves consistently higher Procrustes correlations than both mbVDiT and DeepMicroGen, confirming that imputation preserves the relative positioning of samples in ecological space.

### Per-microbe PCC heatmaps: MAR and MNAR settings

Figures A3 and A4 present per-microbe Pearson correlation coefficients (PCCs) between imputed and ground-truth profiles under the MAR and MNAR missingness settings respectively, complementing the MCAR heatmap presented in Fig. 6. Rows represent individual microbial taxa, sorted by descending mean PCC within each dataset independently; the same row index does not correspond to the same taxon across datasets. The greater heterogeneity observed in the STAD column under MNAR is consistent with the preferential masking of low-abundance taxa in that setting, which presents a more challenging imputation scenario than MAR or MCAR.

### Statistical variance and significance testing for VAE pretraining validation

Table A6 evaluates the performance variance and details statistical significance scores for the VAE pretraining integration across all primary test sets (MCAR setting, 30% missing rate rate). Reported error values highlight SDs across 5 separate model runs. Paired 2-sample *t* test values evaluate the performance delta between configurations, verifying that VAE cross-tissue pretraining produces statistically meaningful execution enhancements across error distributions.

### Data partitioning and leakage prevention

To minimize information leakage, preprocessing and model-dependent steps were carefully separated across data partitions. Prevalence filtering was applied once using a predefined 5% nonzero threshold only to define a common feature space, while TSS normalization was performed independently for each sample using its own row sum, followed by the fixed $\log_{10}\left(\cdot +1\right)$ transformation. The dependency matrices $C_{\mathrm{dir}}$ and $C_{\mathrm{mi}}$ were computed only from the training partition of the target dataset. VAE pretraining excluded the target dataset entirely, and subsequent fine-tuning used only the target training split; validation was used only for hyperparameter selection, and the test set was reserved exclusively for final evaluation. Metadata encoding used a fixed pretrained BERT tokenizer without learning any dataset-specific vocabulary.

#### Case study: Dependency-guided microbial hierarchy

To further investigate the interpretability of the dependency-aware AR ordering, we conducted a case-study analysis on the COAD dataset by visualizing the directed predictive dependency network derived from the DAT dependency mask. Fig. A5 illustrates representative predictive relationships among highly connected microbes, where node colors distinguish early/source-like microbes from late/downstream microbes.

We additionally quantified source-like behavior using the difference between outgoing and incoming dependency counts, defined as out-degree minus in-degree. Microbes with positive source scores act as predictive sources within the learned dependency structure, while microbes with nonpositive scores appear primarily as downstream-dependent taxa.

Although the inferred relationships should not be interpreted as definitive biological causality, these results suggest that the dependency-aware ordering captures biologically plausible microbial dependency organization rather than arbitrary AR permutations.

Overall, this analysis provides additional interpretability evidence supporting the biological relevance of the dependency-guided AR mechanism used in DAT.

### Sensitivity analysis: Dependency network under alternative transformations

Table A7 reports the dependency-network statistics and rank correlations of source-taxon ordering under multiple compositional data transformations on the COAD dataset, using the Log-TSS representation employed in the main experiments as the reference.

All alternative transformations yield positive Spearman correlations relative to the Log-TSS ordering, indicating that the dependency structure identified by the proposed pipeline is not entirely driven by a single normalization choice. Presence–absence binarization shows the strongest agreement with the Log-TSS representation (ρ=0.740), suggesting that portions of the dependency organization captured by the network are preserved even when abundance magnitudes are removed. CLR transformation yields moderate agreement (ρ=0.258), while robust CLR shows weaker but still positive agreement (ρ=0.227). These differences are consistent with the known sensitivity of log-ratio transformations to sparse microbiome data, particularly under high zero inflation (63% sparsity in COAD), where pseudocount handling and geometric mean estimation can substantially alter relative feature geometry [24,25].

Network density remains broadly comparable across transformations. CLR and robust CLR produce 1,391 edges compared to 1,297 under Log-TSS, representing only a moderate increase in connectivity. Although the mutual information thresholds differ substantially across transformations due to scale differences in the transformed representations, the overall dependency organization remains partially consistent.

Importantly, the DAT framework uses the dependency network as an architectural inductive bias for constructing the AR attention mask rather than as a definitive ecological interaction graph. The observed positive rank correlations therefore suggest partial consistency of the dependency-guided ordering used in DAT across multiple compositional representations, while also highlighting that individual dependency edges may vary under alternative data transformations. These results motivate the use of the Log-TSS dependency network for guiding DAT while acknowledging the broader challenges of compositional inference in sparse microbiome data [38,24,25].

### Causal-discovery validation of dependency pairs

To further strengthen the robustness of the directed predictive dependency pairs identified by the cross-sectional conditional association analysis in Predictive Dependency Analysis in Microbiome Data, we applied 3 complementary graphical causal-discovery methods: the PC algorithm, the FCI algorithm, and the GES algorithm. These methods were applied to the COAD dataset after removing near-duplicate highly correlated taxa that caused singular correlation matrices.

The PC algorithm [26] learns a skeleton of the causal graph by iteratively removing edges based on conditional independence tests, producing a completed partially directed acyclic graph. FCI [26] extends PC to allow for latent confounders and selection bias, making it appropriate for observational microbiome data where unmeasured host factors may influence taxon abundances. GES [27] operates in 2 phases, first greedily adding edges to maximize a score function and then removing redundant edges, producing an equivalence class of directed acyclic graphs.

These methods were not used to claim definitive biological causality. Rather, they serve as complementary graphical structure-learning checks to assess whether the strongest predictive dependency pairs identified by the F-statistic analysis are also recovered as statistical adjacencies under observational causal-discovery assumptions. As shown in Fig. A6, all 4 candidate dependency pairs are consistently recovered as adjacencies by all 3 methods (PC, FCI, and GES), confirming that the inferred dependency structure is not an artifact of the F-statistic analysis alone. We interpret this consistency as supporting evidence for the robustness of the directed predictive associations used to construct the DAT attention mask, rather than as proof of directed temporal causality.
